# Controlling Coagulation in Blood with Red Light

**DOI:** 10.1002/anie.202108468

**Published:** 2021-08-31

**Authors:** Patricia Müller, Marlen Sahlbach, Simone Gasper, Günter Mayer, Jens Müller, Bernd Pötzsch, Alexander Heckel

**Affiliations:** ^1^ Goethe University Frankfurt Institute for Organic Chemistry and Chemical Biology Max-von-Laue Str. 9 60438 Frankfurt am Main Germany; ^2^ University Hospital Bonn Institute of Experimental Hematology and Transfusion Medicine Venusberg-Campus 1 53105 Bonn Germany; ^3^ University of Bonn Life and Medical Sciences Institute Center of Aptamer Research & Development Gerhard-Domagk-Str. 1 53121 Bonn Germany

**Keywords:** aptamer, oligonucleotide, photocaging, phototherapeutic window, red light

## Abstract

Precise control of blood clotting and rapid reversal of anticoagulation are essential in many clinical situations. We were successful in modifying a thrombin‐binding aptamer with a red‐light photocleavable linker derived from Cy7 by Cu‐catalyzed Click chemistry. We were able to show that we can successfully deactivate the modified aptamer with red light (660 nm) even in human blood—restoring the blood's natural coagulation capability.

## Graphical abstract

An engineered aptamer with a Cy7‐based photocleavable linker can be used as anticoagulant. With tissue‐penetrating red light, the aptamer is cleaved, and blood clotting is restored in human whole blood.

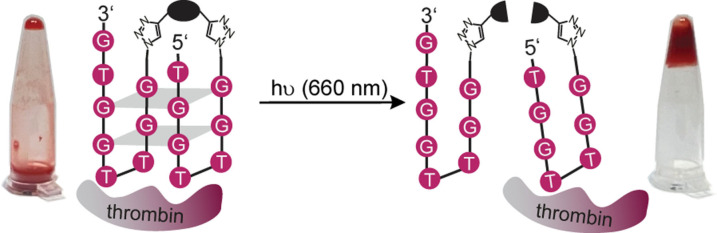

## Introduction

Anticoagulant agents are successfully used in clinical medicine to prevent and treat thromboembolic complications—a major cause of morbidity and mortality in developed countries. Major bleeding is a serious adverse effect of all routinely used anticoagulants. Therefore, there is a clinical need for new anticoagulants allowing rapid reversal of their effect.[^1^, ^2^] Especially, a local antagonization would be desirable, for example if a patient under anticoagulation had to undergo surgery (for example tooth extraction) which can lead to severe bleeding. Up to now the only alternative is to systemically remove the anticoagulation—exposing the patient to a thromboembolic risk over the period of several days.

**Figure 1:**
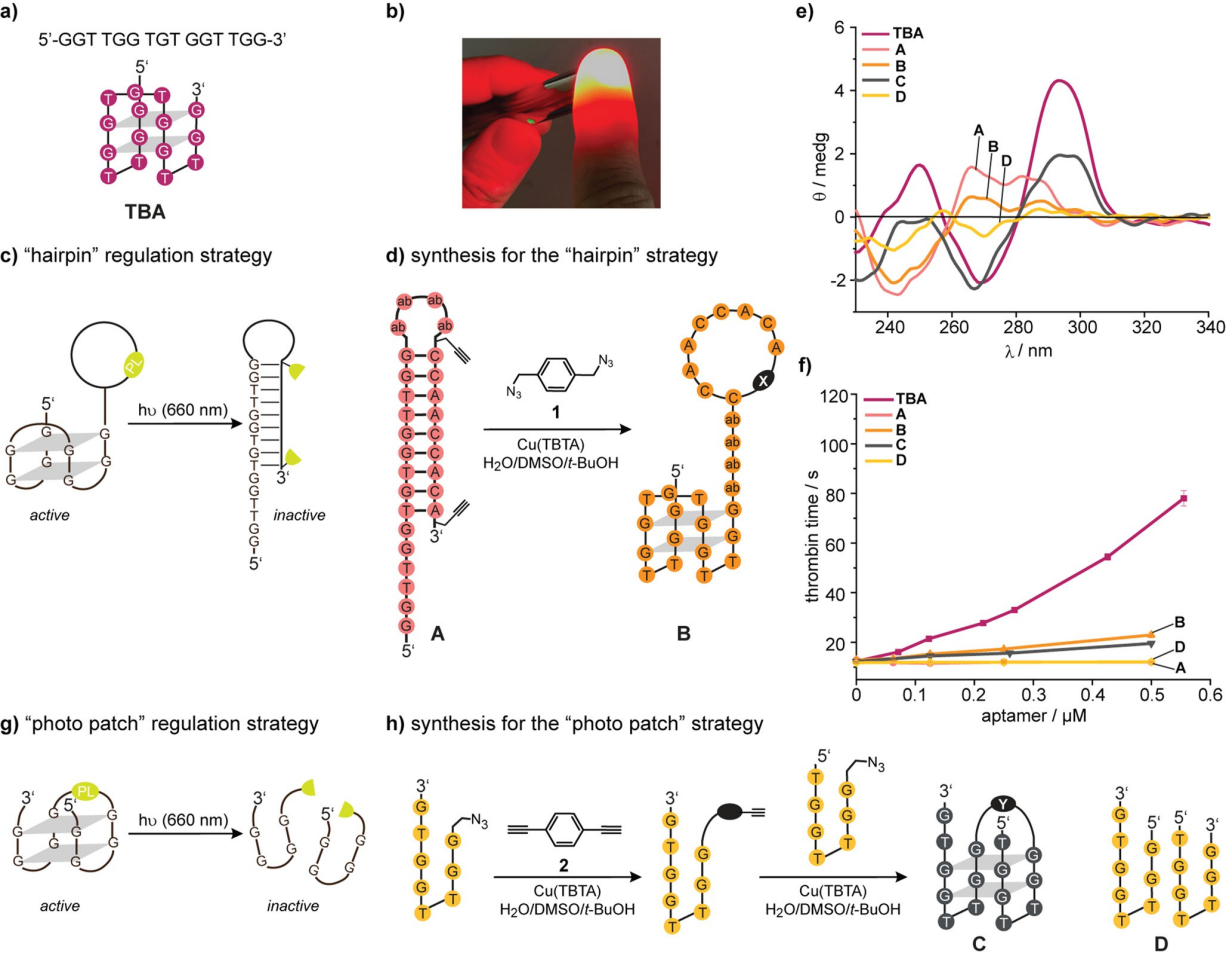
a) The sequence and secondary structure of the thrombin‐binding aptamer (**TBA**). b) Illustration how the light of a red laser pointer (635 nm, 20 mW) can penetrate a finger. c) Initial idea how to deactivate a **TBA**‐derivative with light. d) Synthesis of TBA‐derivatives **A** and **B**, which were used as models to assess the design shown in 1c. e) CD spectra of the indicated oligonucleotides in PBS buffer. f) Results of functional clotting assays (thrombin time [TT]) with the indicated oligonucleotides. g) Second idea how to deactivate a **TBA**‐derivative with light. h) The oligonucleotides **C** and **D** were used as model to assess the design shown in 1e. ab=abasic site, for the structure of the linkers X and Y see Supporting Information.

Over the last twenty years, oligonucleotide therapeutics have shown a remarkable portfolio as orphan drugs for genetic diseases,[^3^, ^4^, ^5^] in n‐of‐1 trials^[6]^ or in the form of aptamers for selectively targeting proteins.^[7]^ Probably the most extensively studied aptamer is the thrombin‐binding aptamer (**TBA**, Figure 1 a). This 15mer DNA oligonucleotide folds into an antiparallel, intramolecular G‐quadruplex structure with a chair‐like conformation.^[8]^ It binds selectively to Exosite I of thrombin and prevents thrombin‐mediated (co)factor‐activation and fibrinogen cleavage, thereby inhibiting blood clotting. **TBA** was the first anticoagulant aptamer agent in phase I clinical trials. In the end, further clinical development was stopped due to unfavorable dosing, high plasma clearance and poor nuclease resistance of **TBA**.^[9]^ By now many studies have addressed these shortcomings and found solutions.[^10^, ^11^, ^12^, ^13^, ^14^, ^15^] A general advantage of therapeutic oligonucleotides is that they can be antagonized by applying complementary antisense oligonucleotides.[^16^, ^17^]

Light is an excellent tool to gain spatio‐temporal control of the function of biomolecules.^[18]^ Several attempts have been described to regulate the function of **TBA** with light: photolabile protecting groups or photoswitches were introduced at specific positions of **TBA** variants that allow either induction or inhibition of its anticoagulant activity.[^19^, ^20^, ^21^, ^22^] For example, *p*‐nitrophenylethyl (NPE) protecting groups were introduced in specific nucleotides to prevent Watson–Crick base‐pairing of **TBA** within an intramolecular antisense strand.[^23^, ^24^] Upon irradiation (*λ*=365 nm), the photolabile groups were released, inducing inactivation of **TBA** by the formation of a hairpin structure. Further attempts have been made with the help of nanomaterials.[^25^, ^26^]

The mentioned photocaging strategies rely on UV‐light or thermal melting caused by high‐power laser irradiation. These conditions are not applicable to in vivo settings as the penetration depth of UV light is very limited. However, within the phototherapeutic window (*λ*=650–850 nm^[27]^) light can penetrate rather deeply (Figure 1 b). Two‐photon irradiation^[28]^ with red light is an interesting technology—but limited to microscopy settings. While it was long considered to be impossible, only recently, one‐photon photolabile protecting groups have been established, which can be cleaved by red or near infrared (NIR)‐light. Lawrence et al. synthesized a photocage derived from a vitamin B12 complex acting as an antenna coupled to the molecule to be released.^[29]^ The groups of Winter, Weinstain and Klán et al. developed a palette of BODIPY‐derived photocages with high quantum yields—cleavable with light ranging from green to NIR.[^30^, ^31^, ^32^, ^33^] Klán et al. could successfully show the activation of BODIPY‐protected carbon monoxide releasing molecules (CORMs) in living mice.^[34]^ In addition, Schnermann et al. successfully introduced heptamethin cyanine (Cy7) as the photocleavable unit for antibody drug conjugates in living mice at 690 nm.^[35]^

None of those red to NIR light photocleavable moieties have been used in the context of light‐regulation of oligonucleotides with therapeutic potential. In the present study, we developed a method to inactivate a **TBA** derivative with red light.

## Results and Discussion

In previous studies, we had demonstrated that the modification of **TBA** is very delicate. Most drastically, any derivatization of the 5′‐end led to a severe loss in **TBA** activity,^[23]^ while modifications at the 3′‐end could even enhance its performance.^[24]^ While G‐quadruplex polymorphism is a likely answer, the exact reason is still unknown. For an efficient inactivation of **TBA** in whole blood with light, we had to both optimize the activity amplitude before and after irradiation and to implement a new red‐light trigger keeping in mind the capricious nature of **TBA**.

At first, we addressed the deactivation strategy separately. Based on our previous results (vide supra), we chose to use 3′‐modifications. Currently the most efficient way to control duplex hybridization is via temporary cyclization.[^32^, ^33^] With very robust design principles, cyclic, “photo‐tethered” oligonucleotides are conformationally unable to form a duplex strand due to the significantly different persistence lengths of a duplex and a single strand. Photo‐tethered oligonucleotides are easy to synthesize and require only one photoactivated bond scission for the full re‐activation of aptamer activity compared to the older method using a heavily modified nucleobase‐caged antisense strand.^[24]^ The first design of this study is shown in Figure 1 c. **TBA** was 3′‐extended with four abasic sites, a 9‐mer antisense region and strategically positioned alkynes (→**A**, Figure 1 d). Using the bisazide **1**, the antisense region was then cyclized in a 1,3‐diploar cycloaddition to obtain the model oligonucleotide **B**. We expected **B** to form an active G‐quadruplex structure and inhibit blood clotting, while **A** should not. However, CD‐spectroscopy (Figure 1 e) suggested that **B** is present in a conformation significantly different from the one of **TBA**. Clotting assays (thrombin time [TT], Figure 1 f) using (colorless) human blood plasma confirmed that **B** is not active enough. This observation is in strong contrast to the previously demonstrated robustness of duplex prevention by cyclization.^[32]^ While the formation of a complete hairpin is impossible, residual single base pairing most likely shifts the delicate G‐quadruplex polymorphism equilibria away from the active conformation. This observation demonstrates once again how complicated the regulation of G‐quadruplex activity can be.

In search for an alternative design principle, we hypothesized that a photolabile linker between the 5′‐ and the 3′‐end of **TBA** could be an alternative to the use of a light‐activated antisense region (Figure 1 g). This design strategy requires the introduction of a strategic nick in the TGT loop, which is known to be variable and not directly involved in thrombin binding.[^36^, ^37^] Following this strategy, however, we expected three new problems: Would the structural requirements for a photocleavable linker be compatible with the aforementioned caveats in 5′/3′‐modification of **TBA**? Would **TBA** tolerate a nick in the TGT loop? Would a complex of thrombin and a photocleaved **TBA** derivative really dissociate under physiological conditions or would the kinetic barrier be too high?

To start assessing these questions, we investigated the model **TBA**‐derivative **C**—prepared using the bisalkyne **2** (Figure 1 h). Synthesis was performed in an optimized two‐step protocol (see also Supporting Information). CD‐spectroscopy suggested that the structure of **C** could indeed be related to the one of **TBA** while the equimolar mixture of the two separate strands (**D**) showed no sign of a G‐quadruplex (Figure 1 e). However, functional plasma‐based TT‐assays revealed a low activity of **C** compared to **TBA** (Figure 1 f).

**Figure 2:**
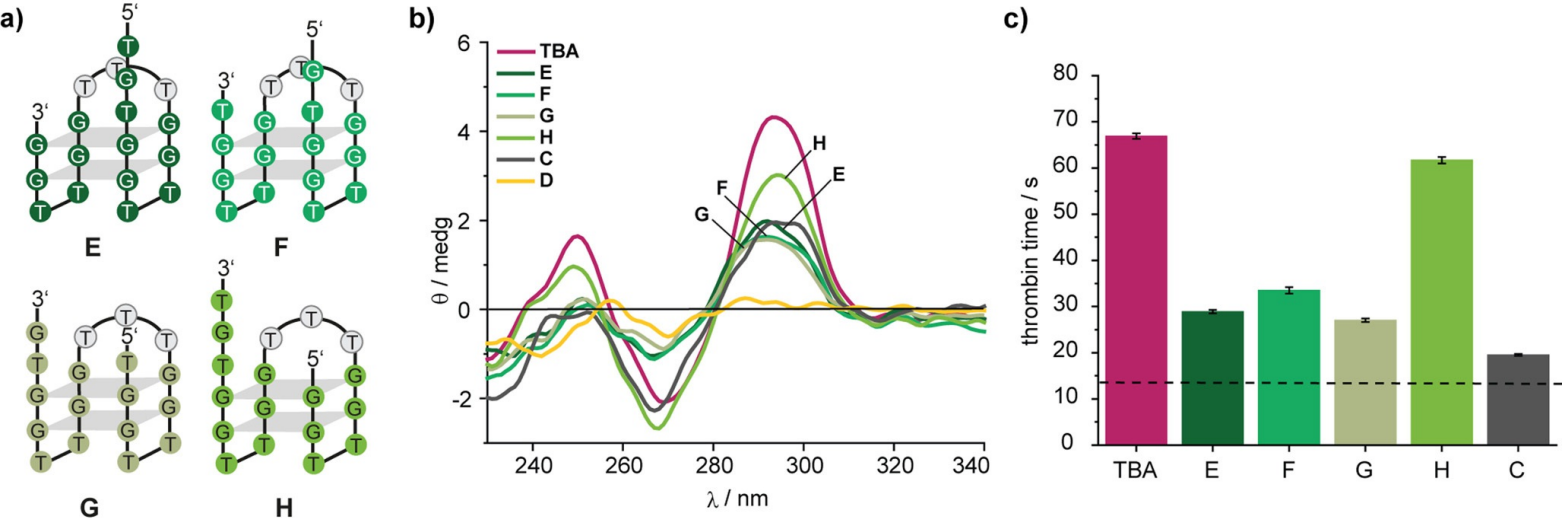
a) Model oligonucleotides **E**‐**H** to investigate the influence of the position of the nick in the design shown in Figure 1 e. b) CD spectra of the indicated oligonucleotides in PBS buffer. c) Results of functional clotting assays (thrombin time [TT]) with the indicated oligonucleotides. The aptamer concentration was 0.5 μM. The dashed line indicates the result in the absence of aptamer.

Assuming that the position of the nick could be important, we continued to screen the **TBA**‐derivatives **E**–**H**—with a TTT linker between the 5′‐ and the 3′‐end instead of the bisalkyne **2** and with nicks at four positions: before, after and within the TGT‐loop (Figure 2 a). CD spectroscopy showed similar folding of **E**, **F**, and **G** (Figure 2 b) very much like the one of **C** (Figure 1 e). In contrast, **TBA**‐variant **H** with a nick after the TGT‐loop showed a CD spectrum, which was more similar to the one of **TBA**. Functional plasma‐based TT‐assays corroborated these observations, in which **TBA**‐variant **H** was found to be the most potent thrombin inhibitor (Figure 2 c) in this set.

**Figure 3:**
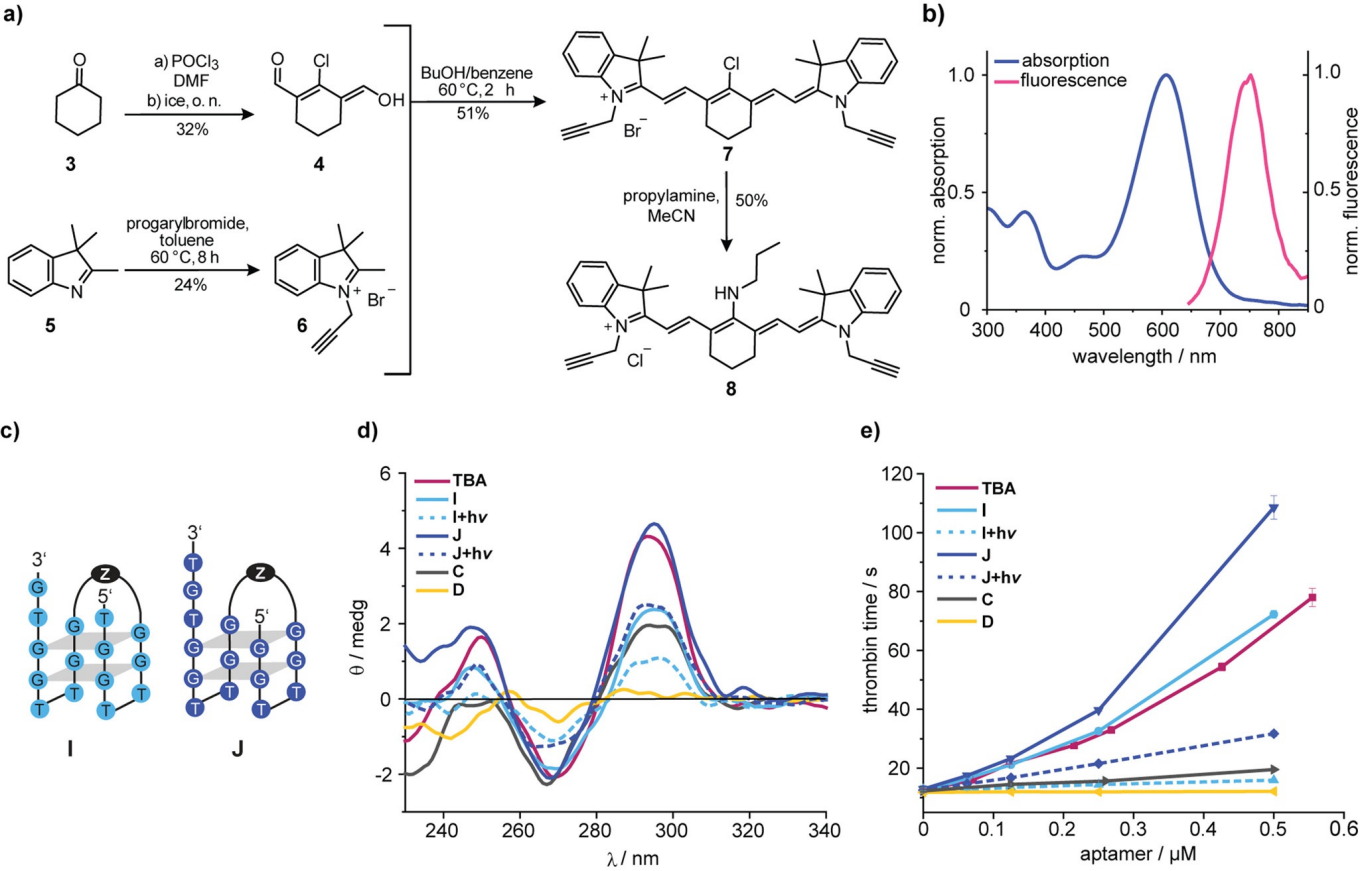
a) Synthesis of the photocleavable Cy7 linker **8**. b) Normalized absorption and fluorescence spectra of the Cy7 linker **8**. c) Sequence of the red‐light cleavable TBA‐derivatives **I** and **J**. For the structure of Z see Supporting Information. d) CD spectra of the indicated oligonucleotides in PBS buffer. e) Results of functional clotting assays (thrombin time [TT]) with the indicated oligonucleotides.

Encouraged by these results, we prepared a photocleavable Cy7 linker **8** (Figure 3 a) in four steps, containing alkynes for a Cu^I^‐catalyzed cycloaddition (for details of the synthesis see Supporting Information). The Cy7‐linker showed the expected photophysical properties compared to previously published analogues^[38]^ and could be quantitatively cleaved at *λ*=660 nm with a quantum yield of 0.12 % (Figure 3 b and Supporting Information). Interestingly, the fluorescence of the Cy7‐chromophore could still be detected in a 96‐well plate in the presence of human blood (Supporting Information). The fact that this linker could therefore also be used for tracking purposes—avoiding an extra fluorophore for this purpose—is noteworthy because incorporation of too many fluorophores can drastically alter the behavior and chemical properties of biomolecules.^[39]^

One has to keep in mind that the spatial requirements for this linker are necessarily different from the ones of the previous optimization. Hence, we had to take the previous results with **H** as optimal compound with a grain of salt for the steps, which were to follow, and designed **TBA**‐derivatives **I** and **J** (Figure 3 c). For illustration purposes, we have depicted the spatial requirements in the Supporting Information.

For the synthesis of **I** and **J** we started with our previously published protocols[^40^, ^41^] to find the right reaction conditions using the photocleavable Cy7‐linker **8** but observed no reaction at all. In contrast, control experiments with a benzylic bisazide‐linker yielded the expected product (data not shown). In a broad screening, we found that the products could be obtained using a combination of high concentrations of reactants (up to 1 mM) and appropriate cosolvent conditions. Surprisingly, the same protocol could be used for an intramolecular cyclization to obtain for example **B**.

To probe the nuclease resistance of our constructs, we incubated **I** in human pooled plasma and analyzed it by PAGE. No degradation products could be observed after 1 h (Supporting Information). **TBA**‐derivative **J** showed a CD‐spectrum, which was almost identical to the one of **TBA**, while the one obtained from **I** was comparable to **H** (Figure 3 d). After irradiation (*λ*=660 nm), significant changes in the CD‐spectra of **I** and **J** were detected. All of the CD spectra were taken in absence of thrombin, which would have made interpretation more difficult. Therefore, thrombin‐induced shifts in G‐quadruplex polymorphisms cannot be detected with these experiments. Functional TT‐assays showed that before irradiation, **J** is even more potent than **TBA** while **I** has a potency similar to **TBA** (Figure 3 e). This shows once more that CD spectroscopy can be used for an initial screening but cannot replace careful coagulation tests for quantitative statements. Most importantly, in both cases, irradiation led to a significant drop of the aptamers’ anticoagulant activity.

It is important to note that up until this stage of the study, for technical purposes, derivatives **I** and **J** had to be irradiated before the addition of thrombin. To address the previously mentioned concern about the kinetic barrier for the dissociation of a thrombin‐aptamer complex after irradiation, we applied a light‐scattering fibrinogen assay. In this assay, a preformed thrombin‐aptamer mixture was incubated with (colorless) purified fibrinogen.

**Figure 4:**
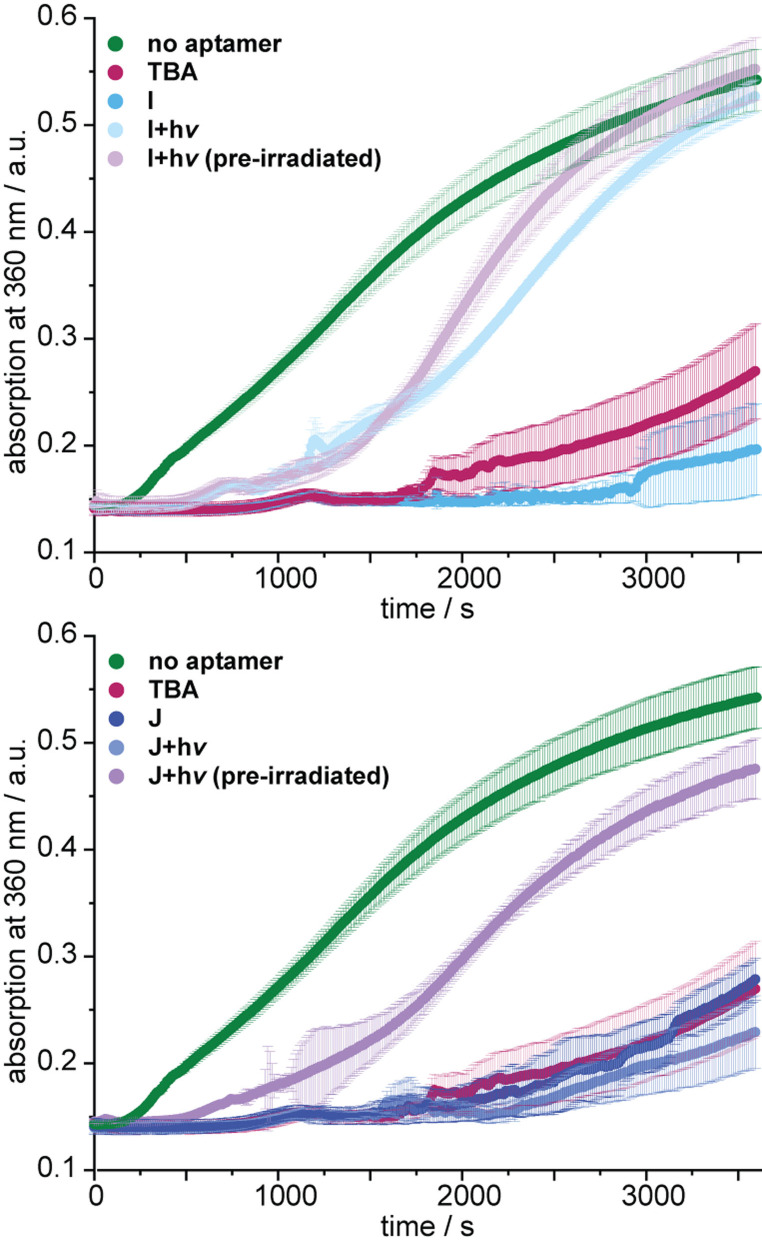
Results of fibrin formation assays using the indicated conditions. A rise in absorbance indicates the presence of active thrombin, which converts fibrinogen to fibrin.

If thrombin is still active in this mixture, fibrinogen is converted to fibrin, which eventually makes the solution opaque. This change can be detected in real‐time by a corresponding increase in absorption. We found that **TBA**‐derivatives **I** and **J** were both inhibiting the conversion of fibrinogen to fibrin—**I** even better than **TBA** (Figure 4). However, upon irradiation with 660 nm, **J** remained inhibitory unless it had been irradiated before the interaction with thrombin. This could speak for the aforementioned kinetic barrier for the dissociation at 37 °C. We were very surprised to see that **I** was not only superior in its activity before irradiation but has apparently a much smaller kinetic barrier, as there was no significant difference between pre‐irradiation and in situ irradiation. This again shows the delicate nature of aptamer‐based regulation of thrombin where subtle changes can have drastic effects on aptamer activity.

**Figure 5:**
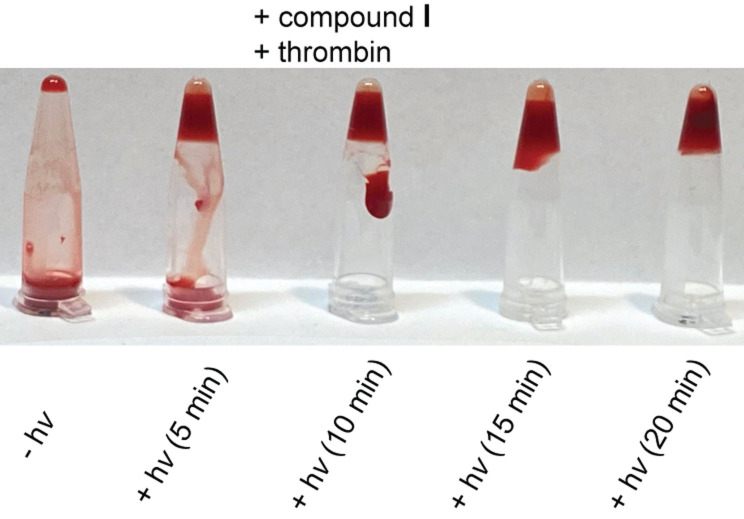
Coagulation tests with human whole blood.

Since both, the plasma‐based TT‐ as well as the fibrinogen assay were performed in the absence of (red) blood cells, we finally went on to test derivative **I** in human whole blood. Without irradiation, in the presence of **I**, we found no signs for thrombin‐mediated coagulation (Figure 5, for more control experiments see Supporting Information). After 5 min of irradiation at 660 nm, coagulation was clearly visible and was almost complete after 10 min of irradiation. To the best of our knowledge, this is the first time that light‐regulation of aptamer activity has been demonstrated in whole blood.

## Conclusion

In summary, we have shown how to engineer a Cy7‐based photocleavage strategy into a “capricious” G‐quadruplex‐based aptamer that affects blood clotting. In contrast to a previous approach, in which thrombin is masked and released by irradiation with UV‐light, the Cy7‐based strategy also enables applications in whole blood. This is of strategic importance *en route* to the spatiotemporal regulation of therapeutic aptamers. The bleeding risk is significantly increased in patients requiring surgical treatment while on anticoagulant therapy. Localized antagonisation of the anticoagulant activity at the wounded area by light would limit blood loss without increasing the risk of thrombosis since the systemic anticoagulant effect remains stable. To the best of our knowledge, our study is the closest study towards the future goal of gaining control of anticoagulant activity with tissue‐penetrating red light.

## Conflict of interest

The authors declare no conflict of interest.

## Supporting information

As a service to our authors and readers, this journal provides supporting information supplied by the authors. Such materials are peer reviewed and may be re‐organized for online delivery, but are not copy‐edited or typeset. Technical support issues arising from supporting information (other than missing files) should be addressed to the authors.

Supporting Information
