# Supplementary material for: Controlling Coagulation in Blood with Red Light
Source: Angew Chem Int Ed Engl. 2021 Aug 31;60(41):22441–6. doi: 10.1002/anie.202108468 (PMC8518524; doi:10.1002/anie.202108468)
Supplement: Supplementary file 1 — Supporting Information [file ANIE-60-22441-s001.pdf]

Angewandte Chemie
Eine Zeitschrift der Gesellschaft Deutscher Chemiker

Supporting Information

# Controlling Coagulation in Blood with Red Light

*Patricia Müller[+], Marlen Sahlbach[+], Simone Gasper, Günter Mayer,* Jens Müller,* Bernd Pötzsch,* and Alexander Heckel**

anie_202108468_sm_miscellaneous_information.pdf

# Table of Contents

Table of Contents ..... 0
1. Chemical Synthesis ..... 1
2. Absorption and fluorescence measurements ..... 2
3. Oligonucleotide synthesis ..... 3
4. General Procedure for NHS-Ester-Labeling ..... 7
5. CuAAC Click Reaction ..... 9
   5.1. Cyclization of A ..... 9
   5.2. Stepwise Conjugation ..... 9
6. Photoproducts ..... 13
7. Irradiation ..... 16
8. CD-spectroscopy ..... 16
9. Actinometry ..... 17
10. Clotting assay to measure the thrombin time in human pool plasma ..... 18
11. Irradiation of the aptamer-thrombin-complex and fibrinogen coagulation assay ..... 18
12. Irradiation in human whole blood and coagulation assay ..... 18
13. Plasma Stability ..... 19
14. Literature ..... 19
15. List of abbreviations ..... 19

# 1. Chemical Synthesis

All reactions were performed under a protective argon atmosphere unless otherwise specified. Reagents and solvents were purchased from commercial sources and used without further purification. Deionised (DI) water was used for all experiments. Reaction progresses were monitored with silica gel 60-coated TLC-sheets and reaction product purifications via column chromatography were performed with silica gel 60, both by Macherey-Nagel. 1H and 13C spectra were recorded on a Bruker AV250, AV400 or AV500 MHz spectrometer. Mass spectra (MS) were obtained using a “Surveyor MSQ” for ESI measurements and high resolution mass spectra (HRMS) were obtained with a ThermoScientific “LTQ Orbitrap XL” (MALDI-HRMS).

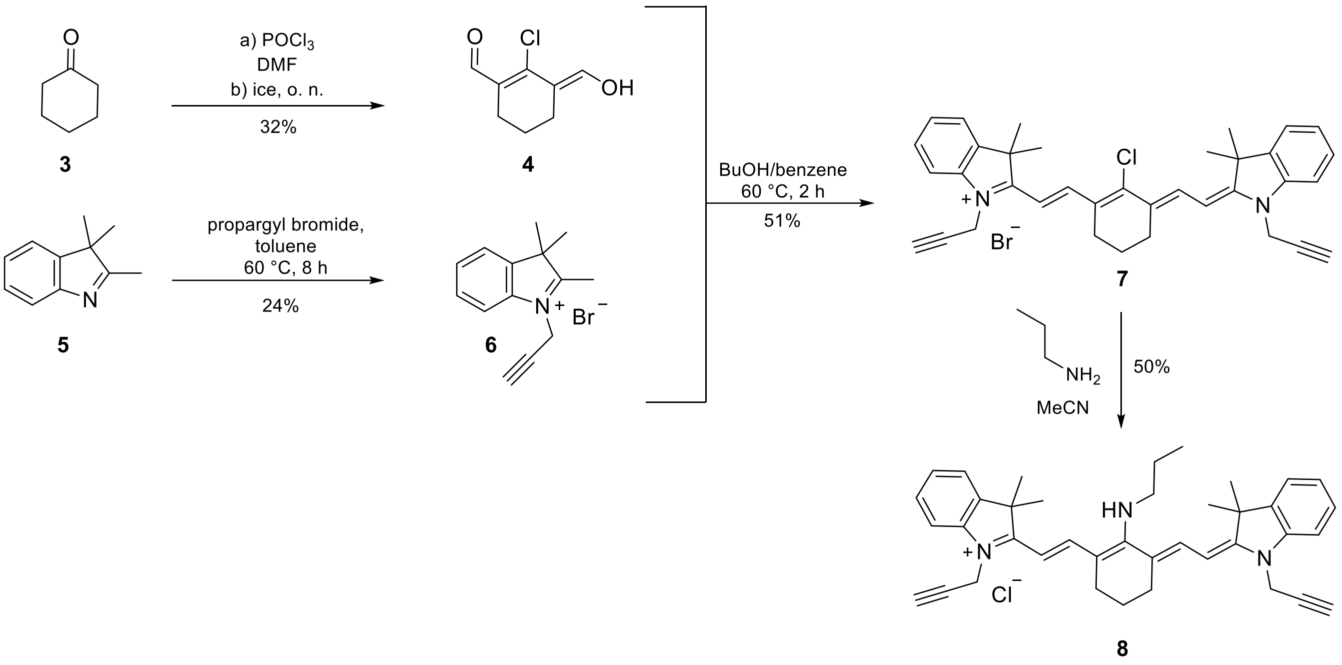


Scheme S1: Synthesis route of the Cy7-linker **8**

**2-Chloro-3-(hydroxymethylene)-1-cyclohex-1-enecarbaldehyde 4**

To 14 ml DMF and 20 ml $CH_2Cl_2$ were mixed and cooled to 0 °C. Within 10 to 15 min 14 ml $POCl_3$ (23.4 g, 153.0 mmol, 3.0 eq.) were added and stirred for 20 min. Next, 5.3 ml cyclohexanone (5.0 g, 51.0 mmol, 1.0 eq.) were added and stirred for 3 h at 80 °C. 50 g ice were added and the hydrolyzed mixture was stored at 8 °C overnight. The crude product was filtered, washed with cold water several times and dried under reduced pressure to obtain crude 4 as an orange powder (2.80 g, 32%). $^1$H-NMR (400 MHz, $CDCl_3$) δ [ppm] = 10.25 (m,1H), 9.76 (s,1H), 3.46-3.44 (t, 1H, *J* = 5 Hz), 2.47-2.45 (t, 4H, *J* = 5 Hz), 1.73 -1.68 (q, 2H, 5 Hz). ESI-MS m/z calcd. for $C_8H_9ClO_2$ 172.61, found $[C_8H_8ClO_2^- \text{-} H^+]$ 170.99.

**2,3,3-Trimethyl-*N*-propargyl-3*H*-indoliumbromide 6**

2 ml of propargyl bromide (20.0 mmol, 80% in toluene, 1.01 eq.) and 3 ml 2,3,3-trimethyl-3H-indole (18.6 mmol, 1.0 eq.) were dissolved in 0.75 mL toluene. The mixture was stirred for 2 h at 60 °C before the solution was stirred at room temperature overnight. Again, the mixture was stirred for another 6 h at 60 °C. The dark purple solid was dried under reduced pressure and purified by column chromatography (cyclohexane:EtOAc 2:1 to CH2Cl2 /MeOH 100:0 to 10:1) to obtain 6 as a colorless foam (1.22 g, 24%). $R_f$ ($CH_2Cl_2$:MeOH =10:1) 0.7. $^1$H-NMR (250 MHz, $CDCl_3$) δ [ppm] = 7.76-7.51 (m, 4H), 5.67 (d, 2H, *J* = 2.5 Hz), 3.20 (s, 3H), 2.57 (t, 1H, *J* = 2.5 Hz), 1.68 (s, 6H). MALDI-HRMS m/z calcd. for $C_{14}H_{16}NBr$ $[M+H]^+$ 279.04457, found $[M+H+^{81}Br]$ 279.09755 (Δm = 0.05298, error 190 ppm).

**2-((*E*)-2-((*E*)-2-chloro-3-(2-((*E*)-3,3-dimethyl-1-(prop-2-yn-1-yl)indolin-2-ylidene)ethylidene)cyclohex-1-en-1-yl)vinyl)-3,3-dimethyl-1-(prop-2-yn-1-yl)-3H-indol-1-ium bromide 7**

365 mg 6 (1.1 mmol, 2.2 eq.) were dissolved in 25 ml butanol:benzene (7:3). 88 mg 4 (0.5 mmol, 1.0 eq.) were added and stirred for 2 h at 60 °C. The solvent was removed under reduced pressure and purified by column chromatography ($CH_2Cl_2$:MeOH = 100:0 to 10:1) to obtain 7 as a green-metallic solid (170 mg, 51%). $R_f$ ($CH_2Cl_2$:MeOH =10:1) 0.6. $^1$H-NMR (400 MHz, $CDCl_3$) δ [ppm] = 8.35 (d, 2H, *J* = 14 Hz), 7.42-7.37 (q, 4H, *J* = 7.8 Hz), 7.27-7.25 (m, 4H), 6.43 (d, 2H, *J* = 14 Hz), 5.12 (d, 4H, *J* = 2.4 Hz), 2.79 (t, 4H, *J* = 6 Hz), 2.40 (t, 2H, *J* = 1.6 Hz), 1.98 (t, 2H, *J* = 6 Hz), 1.74 (s, 12 H). ESI-MS m/z calcd. for $C_{36}H_{36}N_2ClBr$ 610.17 $[M\text{-}^{81}Br^-]^+$ 531.30.

**2-((*E*)-2-((*E*)-3-(2-((*E*)-3,3-dimethyl-1-(prop-2-yn-1-yl)indolin-2-ylidene)ethylidene)-2-(propylamino)cyclohex-1-en-1-yl)vinyl)-3,3-dimethyl-1-(prop-2-yn-1-yl)-3H-indol-1-ium 8**

142 mg 7 (0.23 mmol, 1.00 eq.) were dissolved in 30 ml MeCN in the absence of light. 0.08 ml propylamine (57.9 mg, 0.98 mmol, 10 eq.) were added and the mixture was stirred for 1.5 h at 50 °C. The solvent was removed under reduced pressure and purified by column chromatography ($CH_2Cl_2$:MeOH = 100:0 to $CH_2Cl_2$:MeOH = 95:5) to obtain 8 as a blue-metallic solid (75.2 mg, 50%). $R_f$ ($CH_2Cl_2$:MeOH =10:1) 0.5. $^1$H-NMR (500 MHz, $CDCl_3$) δ [ppm] 7.70 (d, 2H, *J* = 3.0 Hz), 7.17 (d, 4H, *J* = 3.0 Hz=, 7.04 (t, 4H, *J* = 7.4 Hz), 6.85 (d, 2H, *J* = 7.7 Hz), 6.11 (d, 1H, *J* = 6.2 Hz), 5.63 (d, 2H, 12.4 Hz), 4.48 (s, 2H), 3.87 (s, 4H), 2.56 -2.52 (m, 4H), 2.28 (s, 2H), 2.08 (dd, 2H, $^2J$ = 14.5 Hz, *J* = 7.0 Hz), 1.73 (s, 12 H), 0.96 (t, 3H, *J* = 5.7 Hz).$^{13}$C (125.8 MHz, $CDCl_3$) δ [ppm] 172.1, 165.0, 142.5, 140.0, 136.5, 128.1, 122.3, 107.7, 94.4, 72.78, 53.6, 51.6, 47.4, 42.8, 41.1, 37.5, 37.2, 33.8, 32.7, 32.1, 29.8, 29.1, 25.9, 24.1, 22.8, 21.3, 14.2, 11.4). MALDI-MS m/z calcd. for $C_{39}H_{44}N_3Cl$ [M-Cl$^-$]$^+$ 544.35352 found 554.35181 (Δm = 0.00171 = 2.7 ppm).

## 2. Absorption and fluorescence measurements

The UV/vis absorption spectrum of the Cy7-linker was recorded on an Evolution 300 UV/vis spectrometer at 25 °C in a 1 cm path length cuvette in MeOH/PBS (1:2).

The extinction coefficient of the Cy7-linker in MeOH/PBS (1:2) at 660 nm is 4470 $M^{-1}$ $cm^{-1}$.

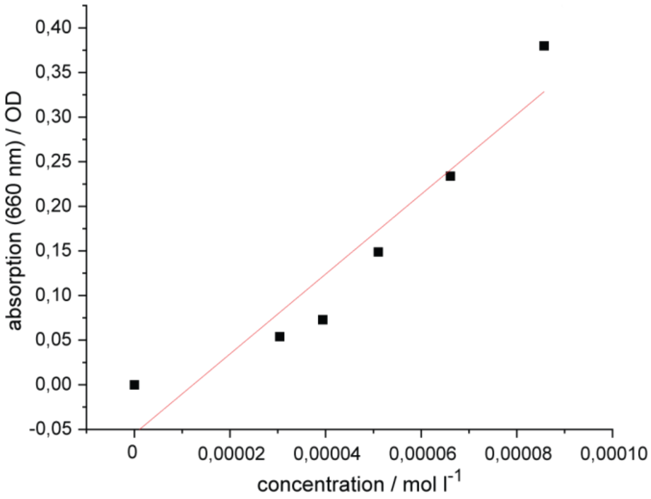


Figure S1: Plot of the concentration versus absorption at 660 nm to determine the extinction coefficient of the Cy7 linker at 660 nm in MeOH:PBS (1:2).

The fluorescence spectrum of the Cy7-linker was recorded on a Tecan Infinite M200 Pro plate reader in methanol (excitation wavelength 600 nm). To measure the fluorescence of the Cy7-linker in human whole blood, 16 µl of a 2 µM aqueous solution of oligonucleotide **K** (see also section 9. actinometry) were mixed with 64 µl human whole blood in a 96 well plate.

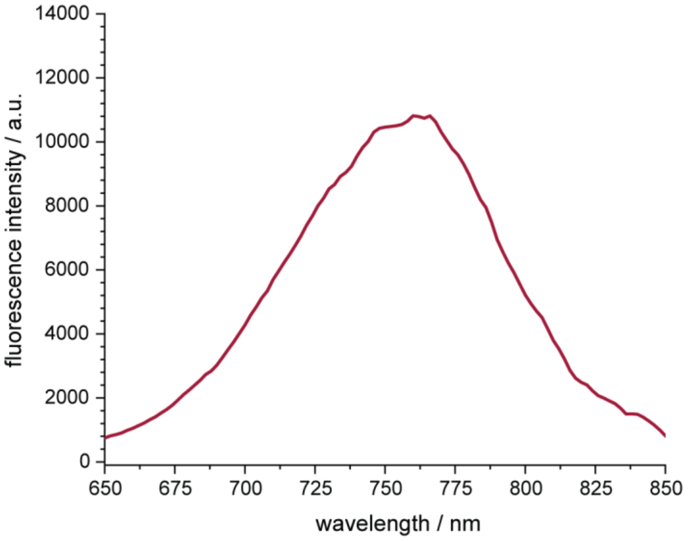


Figure S2: Fluorescence spectrum of the Cy7-linker in human whole blood.

To illustrate the decrease in fluorescence of the Cy7-linker by irradiation with red light (660 nm), 3 µM in 1xPBS of **K** were irradiated at 37 °C with a M660L4 LED (*Thorlabs*, 660 nm, operated at 1000 mA, 140 mW) using a *Thorlabs* LED driver DC4104 with DC4100-HUB and a ACL 2520-A lens (*Thorlabs*) in a cuvette (56 µl, d = 1 cm) and durations of 0-540s. This solution was transferred to a 96 well plate, made up to 100 µl and then the fluorescence was measured (excitation wavelength 605 nm).

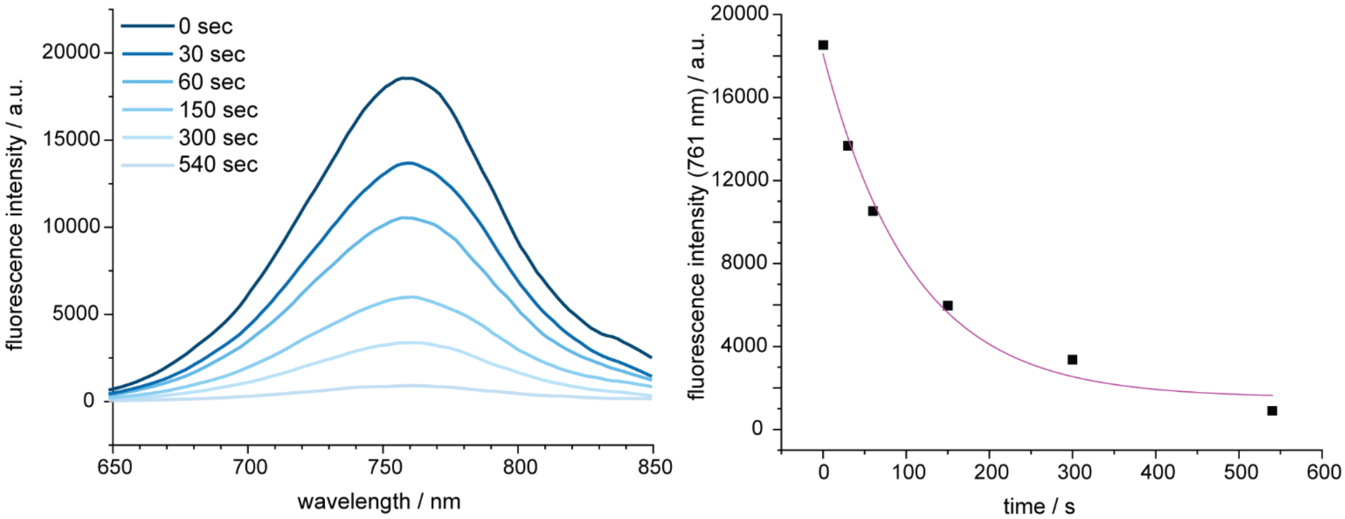


Figure S3: Decrease in fluorescence of Cy7-linker as a function of irradiation time.

## 3. Oligonucleotide synthesis

The used Milli-Q water was treated with DEPC (0.1%) overnight and autoclaved before usage.

The following oligonucleotides were synthesized by solid-phase synthesis:

| | |
|---|---|
| **A** | 5‘-GGT TGG TGT GGT TGG (**ab**)(**ab**)(**ab**)(**ab**) **m**$^1$ CCA ACC ACA **m**$^1$-3‘ |
| **i-1** | 5‘-**m**$^3$-GGT TGG TG-3‘ |
| **j-1** | 5‘-**m**$^3$-GGT TGG TGT-3‘ |

The following oligonucleotides were purchased HPLC purified from *Biomers*:

| | |
|---|---|
| **i-2** | 5‘-T GGT TGG **m**$^2$-3‘ |
| **j-2** | 5‘-GGT TGG **m**$^2$-3‘ |
| **TBA** | 5‘-GGT TGG TGT GGT TGG-3‘ |
| **E** | 5‘-TGT GGT TGG TTT GGT TGG-3‘ |
| **F** | 5‘-GT GGT TGG TTT GGT TGG T-3‘ |
| **G** | 5‘-T GGT TGG TTT GGT TGG TG-3‘ |
| **H** | 5‘-GGT TGG TTT GGT TGG TGT-3‘ |

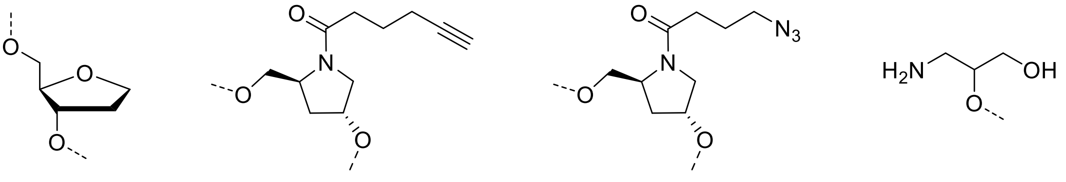


**ab** **m**$^1$ **m**$^2$ **m**$^3$

Figure S4: Chemical structures of the modifier used for the oligonucleotide synthesis.

**D** is the equimolar solution of **i-1** and **i-2.**

Phosphoramidite used for solid phase synthesis:

| | |
|---|---|
| G: | *i*Pr-Pac-dG-CE phosphoramidite (*Linktech*) or dG(n-*t*BPAC) CED phosphoramidite (*ChemGenes)* |
| T: | DMT-dT phosphoramidite (*Sigma Aldrich*) or dT-CE phosphoramidite (*Linktech*) |
| C: | DMT-dC(ac) phosphoramidite (*Sigma Aldrich*) |
| A: | DMT-dA(tac) phosphoramidite (*Sigma Aldrich*) |
| $m^1$ | Alkyne amidite hydroxyprolinol (*Lumiprobe*) |
| $m^3$ | Fmoc-Amino-DMT C3 CED phosphoramidte (*ChemGenes*) |
| ab: | dSpacer-CE phosphoramidte (*Linktech*) |

Solid phase synthesis of the alkyne-modified DNA oligonucleotide **A** was performed on ABI 392 DNA/RNA synthesizer carried out on a 1 µmol scale. Alkyne CPG 1000 Å (Lumiprobe) was used as solid support. The oligonucleotides were synthesized in DMT-ON mode under UltraMILD© conditions (Pac-Anhydride/pyridine in tetrahydrofuan as capping reagent). 0.3 M BTT in acetonitrile (*emp Biotech*) was used as activator. The coupling time was 34 s. After deprotection with 32% $NH_3$ (4 h, 28 °C) the solvent was evaporated under reduced pressure in a vacuum centrifuge a at 4 °C and the crude product was purified by RP-HPLC on a *Agilent 1200* equipped with a *Waters XBridge Peptide BEH C18 OBD Prep* column (300 Å, 5 µm, 10 mm x 250 mm) using gradient 1 (400 mM HFIP/16.3 mM TEA buffer pH 7.9, methanol, gradient 1: 5% MeOH for 2 min, 5% to 25% MeOH in 2 min, 25% to 50% MeOH in 7 min, 50% to 100% MeOH in 2 min, flow rate 4 ml/min, 35 °C). After evaporation of the solvent the DMT group was removed with 80% acetic acid (20 min, 25 °C) and the oligonucleotide was purified again by RP-HPLC on a *Agilent 1200* equipped with a *Waters XBridge Peptide BEH C18 OBD Prep* column (300 Å, 5 µm, 10 mm x 250 mm) using gradient 1.

Solid phase synthesis of the 5'-amino-modified DNA oligonucleotides (**i-1** and **j-1**) were performed on a *Äkta OligoPilot 10* synthesizer carried out on a 14 µmol scale. Universal support (Universal CPG 1000/110 S, *Linktech*) was used as solid support. The oligonucleotides were synthesized in DMT-OFF mode under UltraMILD© conditions (Tac-Anhydride/pyridine in acetonitril as capping reagent). 0.3 M BTT in acetonitrile (*Sigma Aldrich*) was used as activator (60%). Phosphoramidites were desolved 0.1 M in acetonitrile and per coupling 3 eq were used, with a recycle time of 3 min. The cyanoethyl groups were removed by flushing the solid phase column with 3 ml 20% diethylamine (DEA) in MeCN for 10 min, followed by washing with MeCN and drying in vacuum. After deprotection with 32% $NH_3$ (5-20 min, 130 °C, microwave reactor (Initiator Biotage)) the solvent was evaporated under reduced pressure in a vacuum centrifuge at 4 °C. The crude product was purified by AEX-HPLC on a *Äkta Purifier* equipped with a *Cytiva Resource Q 6 ml* column using gradient 2 (buffer A:10 mM Tris-HCl/ 25 mM $NaClO_4$/ with 20% MeCN buffer pH 8, buffer B: 10 mM Tris-HCl/ 500 mM $NaClO_4$/ with 20% MeCN buffer pH 8, gradient 2: 0% to 30% buffer B in 20 min, flow rate 6 ml/min). Then, the oligonucleotide was desalted by RP-HPLC on a *Äkta Purifier* equipped with a *Waters XBridge Peptide BEH C18 OBD Prep* column (130 Å, 5 µm, 10 mm x 50 mm) using gradient 3 (400 mM HFIP/16.3 mM TEA buffer pH 7.9, methanol, gradient 3: 5% MeOH for 8 min, 5% to 100% MeOH in 8 min, 100% MeOH for 12 min, flow rate 4 ml/min).

The purity and identity was confirmed by LC-MS (LC-system: *Agilent 1200* equipped *Waters XBridge Peptide BEH C18* column (300 Å, 3.5 µm, 2.1 mm x 250 mm), buffers: 400 mM HFIP/16.3 mM TEA buffer pH 7.9, methanol; MS-system: *Bruker micrOTOF-QII ESI).*The LC gradient used is listed at the respective position.

Table S1 : ESI-MS data of the synthesized and purchased DNA oligonucleotides.

| ID | mass calculated [Da] | mass found [Da] |
|---|---|---|
| **A** | 8687.6 | 8687.7 |
| **i-1** | 2648.5 | 2648.8 |
| **j-1** | 2952.5 | 2952.9 |
| **i-2** | 2456.5 | 2456.8 |
| **j-2** | 2152.4 | 2152.7 |
| **TBA** | 4723.8 | 4724.4 |
| **E** | 5636.0 | 5636.7 |
| **F** | 5636.0 | 5636.7 |
| **G** | 5636.0 | 5636.7 |
| **H** | 5636.0 | 5636.7 |

compound **A**

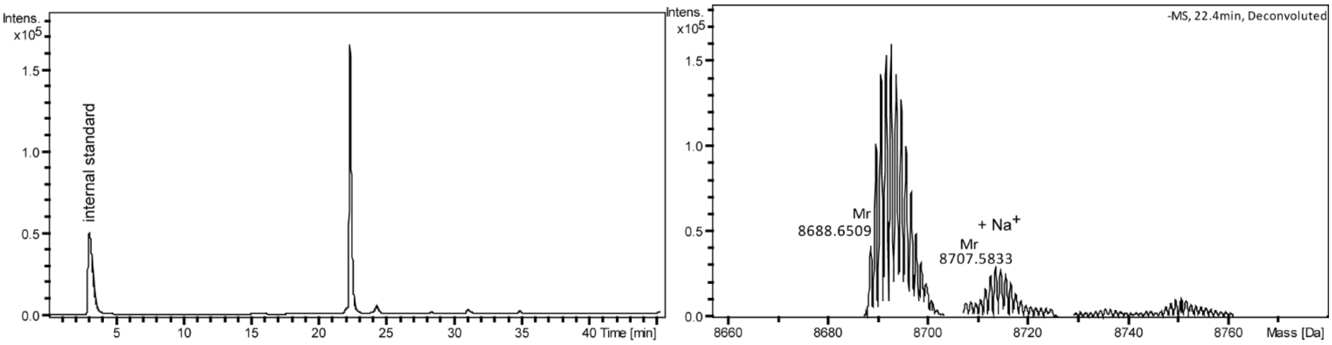


compound **i-1**

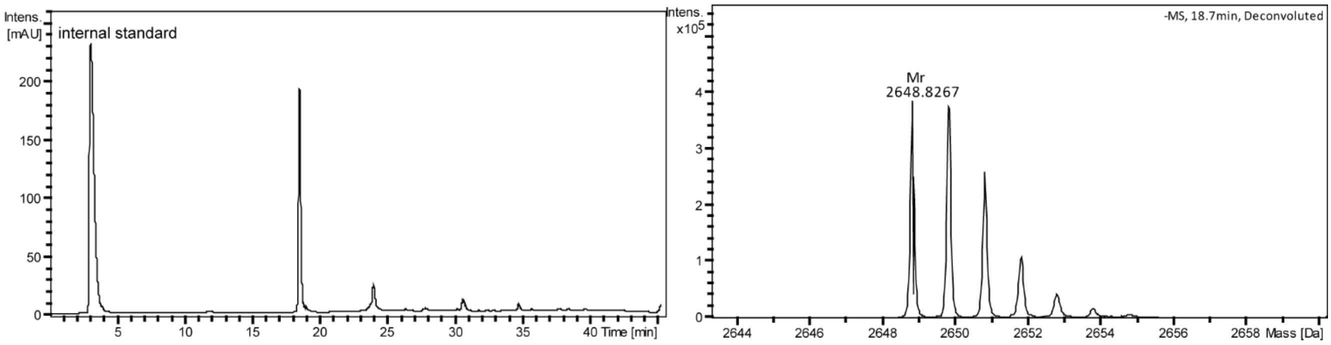


compound **j-1**

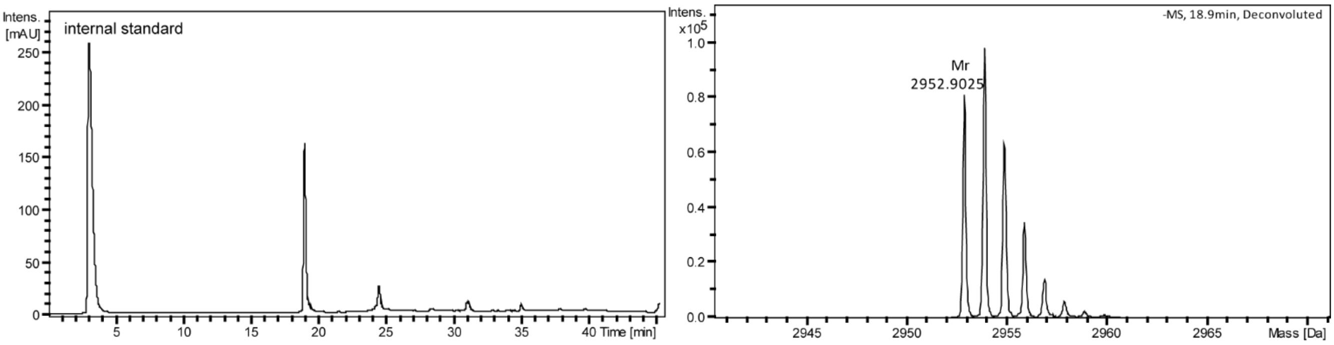

compound **i-2**

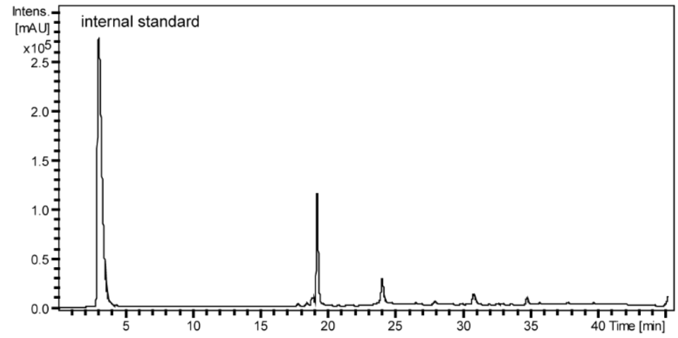


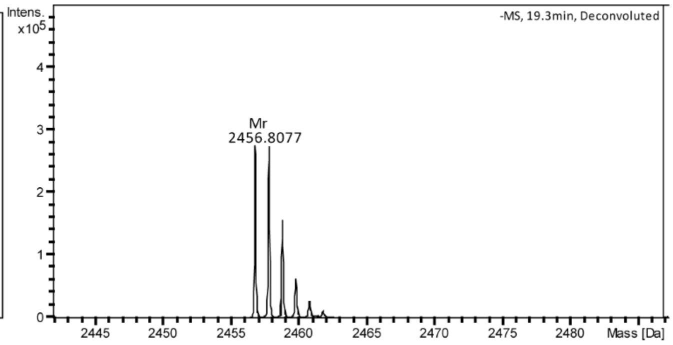


compound **j-2**

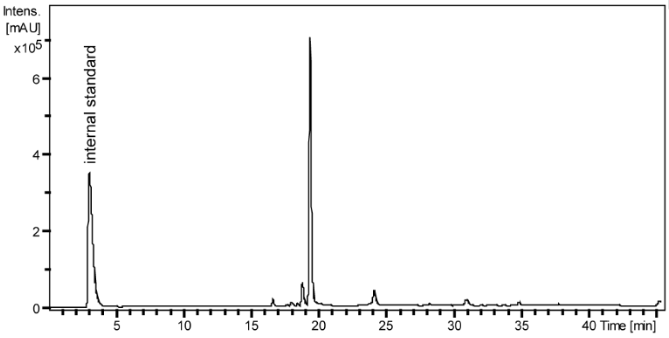


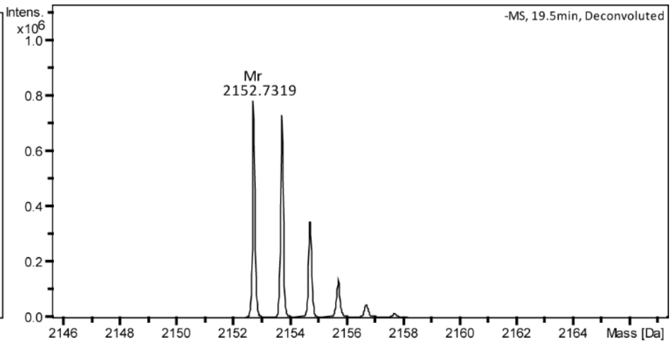


compound **TBA**

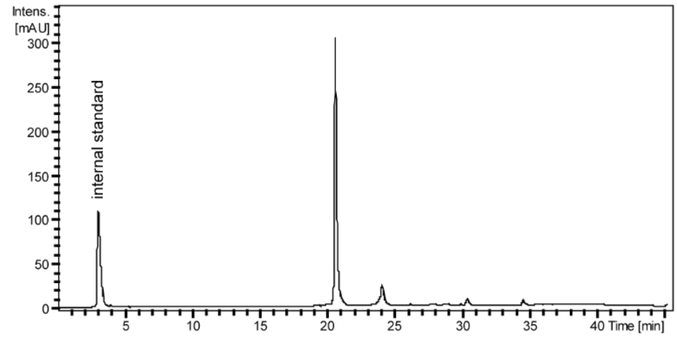


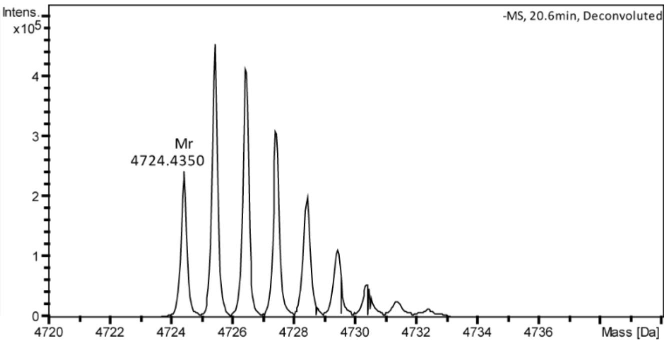


compound **E**

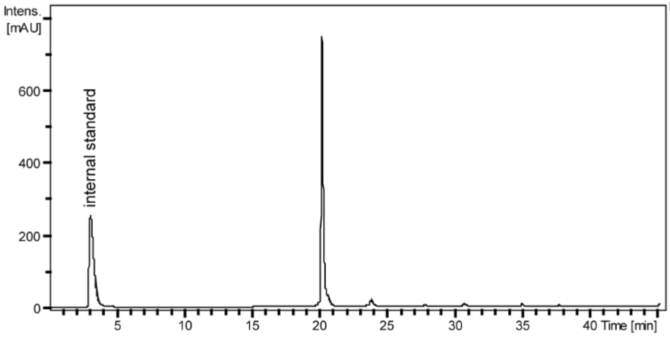


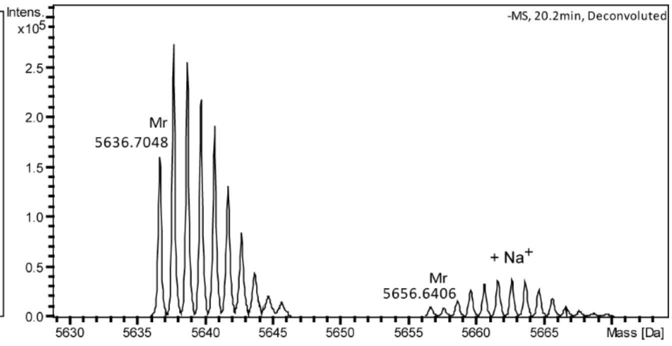

compound **F**

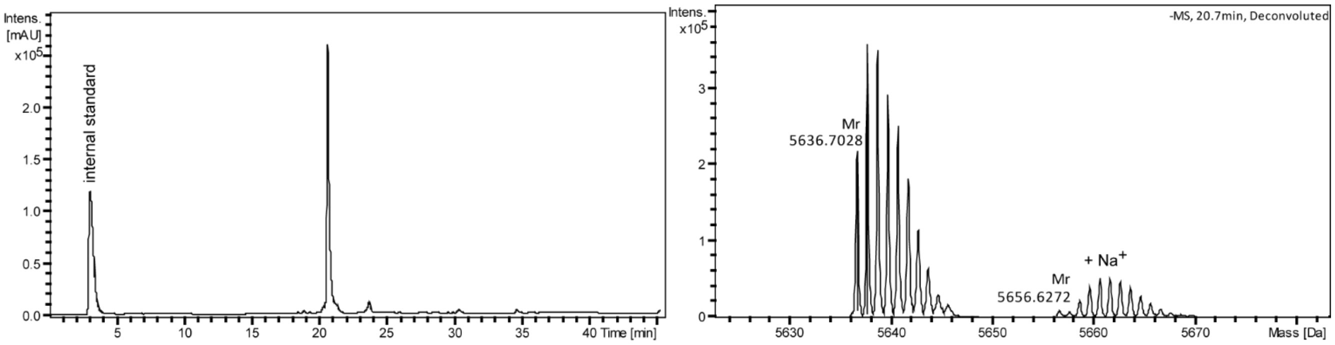


compound **G**

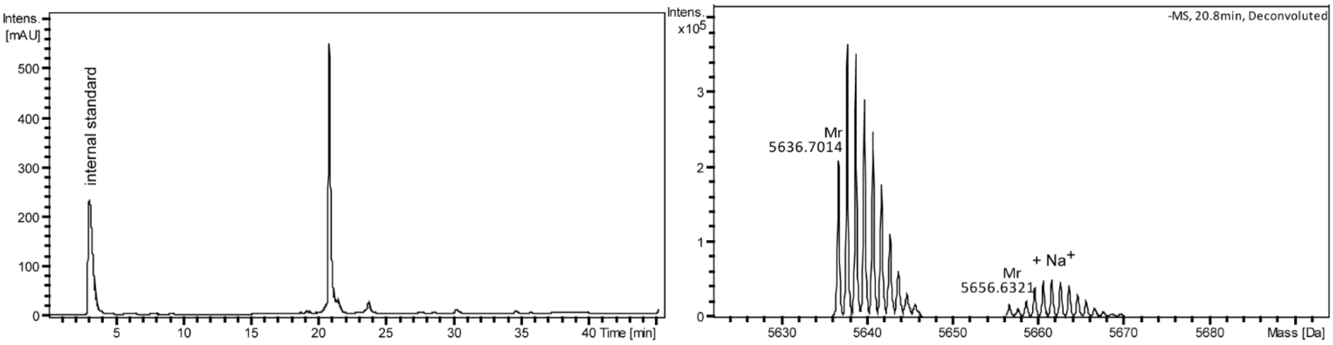


compound **H**

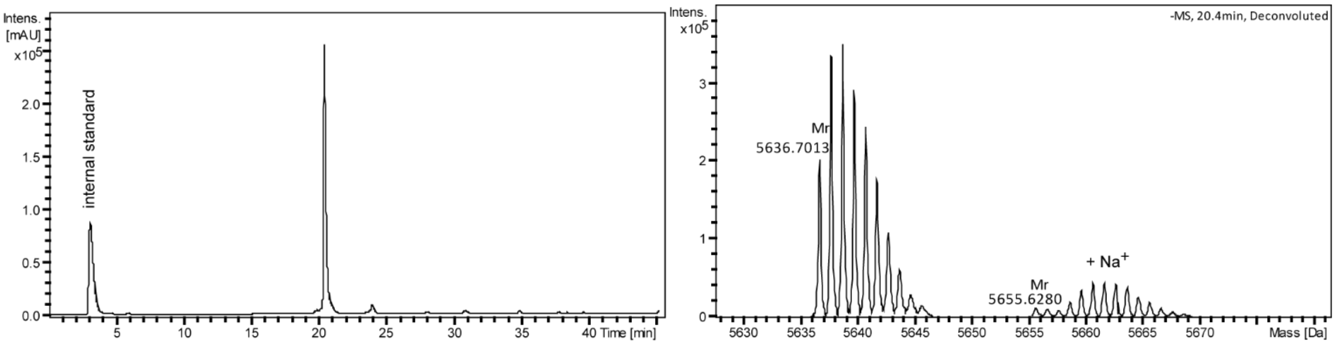


Figure S5: LC-spectrum (left side) and deconvoluted ESI-LC/MS spectrum (right side) of the respective synthesized and purchased compounds. (LC gradient: 5% MeOH for 5 min, 5% to 30% MeOH in 2 min, 30% to 80% MeOH in 20 min, 80% to 100% MeOH in 5 min, 100% MeOH for 5 min, 100% to 5% MeOH in 5 min, 5% MeOH for 3 min, flow rate 0.25 ml/min, 40 °C).

## 4. General Procedure for NHS-Ester-Labeling

To introduce the azide functionality required for the CuAAC click reaction, the 5'-amino-modified oligonucleotide **i-1** or **j-1** (10 nmol) was dissolved in 24 µl borate buffer (stocksolution A) followed by addition of 4 µl azidobutyric acid NHS ester solution B (0.169 m in MeCn, 67 eq). The reaction tube was shaked at 25 °C overnight. The mixture was diluted in Milli-Q water and purified by RP-HPLC on a *Agilent 1200* equipped with a *Waters XBridge Peptide BEH C18 OBD Prep* column (130 Å, 5 µm, 10 mm x 50 mm) using gradient 4 (400 mM HFIP/16.3 mM TEA buffer pH 7.9, methanol, gradient 4: 5% MeOH for 2 min, 5% to 25% MeOH in 2 min, 25% to 50% MeOH in 7 min, 50% to 100% MeOH in 2 min, flow rate 4 ml/min, 30 °C)

Stocksolution A: $Na_2B_4O_7$ (100 mM in $H_2O$, pH 8.4)
Stocksolution B: Azidobutyric acid NHS ester (0.169 M in MeCN)

Table S2: ESI-MS data of the azide labeled oligonucleotides.

| ID | sequence | mass calculated [Da] | mass found [Da] |
|---|---|---|---|
| **i-1-azide** | 5’-**m4** GGT TGG TG-3’ | 2760.5 | 2759.9 |
| **j-1-azide** | 5’-**m4** GGT TGG TGT-3’ | 3064.6 | 3064.0 |

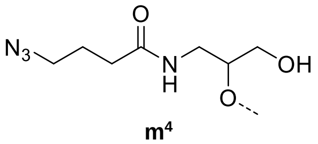


**m4**

Figure S6: Chemical structure of the azide-labeled amino modifier **m3.**

compound **i-1-azide**

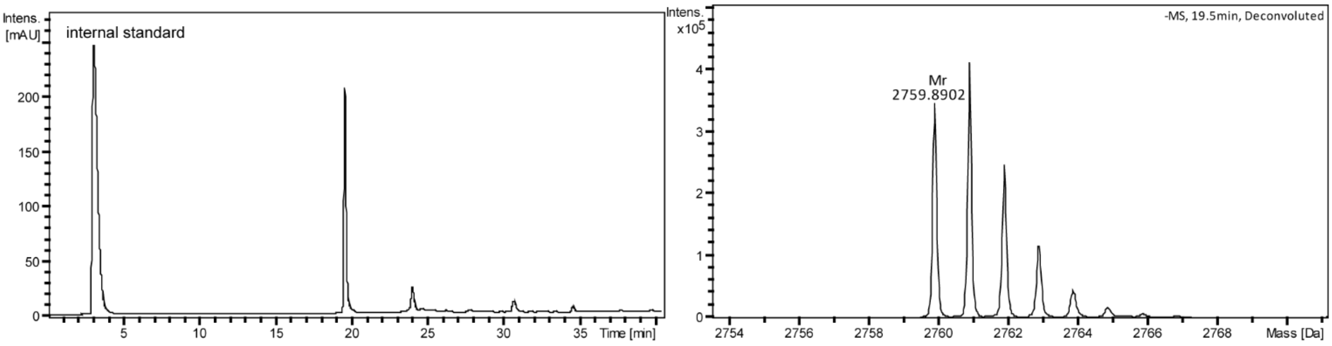


compound **j-2-azide**

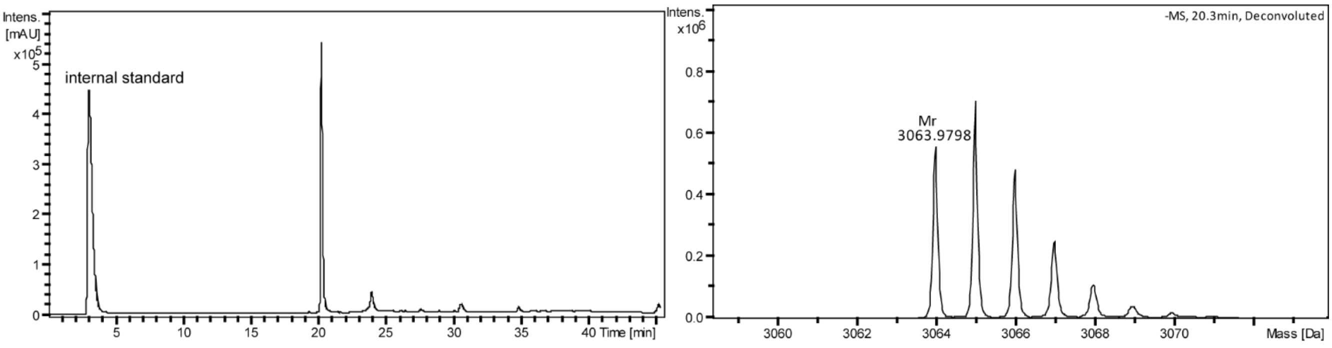


Figure S7: LC-spectrum (left side) and deconvoluted ESI-LC/MS spectrum (right side) of the respective azide-labeled compounds **i-2-azide** and **j-2-azide**. (LC gradient: 5% MeOH for 5 min, 5% to 30% MeOH in 2 min, 30% to 80% MeOH in 20 min, 80% to 100% MeOH in 5 min, 100% MeOH for 5 min, 100% to 5% MeOH in 5 min, 5% MeOH for 3 min, flow rate 0.25 ml/min, 40 °C).

# 5. CuAAC Click Reaction

## 5.1. Cyclization of A

For preparation of **B**, to an aqueous solution of **A** (5 nmol in 2.5 µl Milli-Q water, final conc. 2 mM) were added in following order: bis-azido-solution A1 (0.55 µl, 1.1 eq), freshly prepared Cu(I)TBTA-complex solution B (1.5 µl) and DMSO to reach a final oligonucleotide concentration of 770 µM. The reaction tube was sealed under argon and shaken for 180 min at 60 °C. The reaction mixture was diluted in Milli-Q water and purified by RP-HPLC on a *Agilent 1200* equipped with a *Waters XBridge Peptide BEH C18* column (300 Å, 3.5 µm, 4.6 mm x 250 mm) using gradient 5 (400 mM HFIP/16.3 mM TEA buffer pH 7.9, methanol, gradient 5: 5% MeOH for 2 min, 5% to 25% MeOH in 3 min, 25% MeOH for 6 min, 25% to 40% MeOH in 15 min, 40% to 100% MeOH in 6 min, flow rate 0.7 ml/min, 60 °C)

## 5.2. Stepwise Conjugation

*First step: conjugation of the bis-alkyne-Linker **2** to the first aptamer part*
**i-2** (10 nmol) was dissolved in 5 µl 1x PBS (with additional 50 mM KCl) to reach a final concentration of 2 mM. To this were added in following order: bis-alkyne-linker-solution A2 (4 µl, 4 eq), Cu(I)TBTA-complex solution B (3 µl) and DMSO to reach a final oligonucleotide concentration of 690 µM. The reaction tube was sealed under argon and shaken for 180 min at 45 °C. The reaction mixture was diluted in Millli-Q water and purified by RP-HPLC on a *Agilent 1200* equipped with a *Waters XBridge Peptide BEH C18* column (300 Å, 3.5 µm, 4.6 mm x 250 mm) using gradient 6 (400 mM HFIP/16.3 mM TEA buffer pH 7.9, methanol, gradient 6: 5% MeOH for 2 min, 5% to 25% MeOH in 3 min, 25% to 45% MeOH in 20 min, 45% to 100% MeOH in 3 min, flow rate 0.7 ml/min, 30 °C).

*First step: conjugation of the Cy7 linker **8** to the first aptamer part*
**i-2** or **j-1-azide** (10 nmol) were dissolved in 5 µl 1x PBS (with additional 50 mM KCl) to reach a final oligonucleotide concentration of 2 mM. To this solution were added in following order: Cy7-linker-solution A3 (7 µl, 7 eq), freshly prepared Cu(I)TBTA-complex solution B (3 µl) and DMSO to reach a final oligonucleotide concentration of 625 µM. The reaction tube was sealed under argon and shaken for 130 min at 25 °C. The reaction mixture was diluted in Milli-Q water and purified by RP-HPLC on a *Agilent 1200* equipped with a *Waters XBridge Peptide BEH C18* column (300 Å, 3.5 µm, 4.6 mm x 250 mm) using gradient 7 (400 mM HFIP/16.3 mM TEA buffer pH 7.9, methanol, gradient 7: 5% MeOH for 2 min, 5% to 30% MeOH in 3 min, 30% to 40% MeOH in 2 min, 40% to 45% MeOH in 3 min, 45% to 80% MeOH in 17.5 min, 80% to 100% MeOH in 2.5 min, flow rate 0.7 ml/min, 30 °C).

*Second step: conjugation of the second aptamer part*
The first aptamer part (5 nmol, 1 eq), which is already linked to the Cy7- or to the bis-alkyne-linker, and the second oligonucleotide (1.5 eq) with a free azide group were dissolved in 1x PBS (with additional 50 mM KCl) to reach a concentration of 1.67 mM with respect to the first oligonucleotide. After, 3.5 µl DMSO and the Cu(I)TBTA-complex solution B (1.5 µl) were added. The reaction tube was sealed under argon and shaken for 180 min at 30 °C. The reaction mixture was diluted in Milli-Q water and purified by RP-HPLC on a *Agilent 1200* equipped with a *Waters XBridge Peptide BEH C18* column (300 Å, 3.5 µm, 4.6 mm x 250 mm) using gradient 6 (conjugated via the bis-alkyne-linker) or gradient 7 (conjugated via the Cy7-linker).

Stock solution A1: Bis-azido-solution (1,4-Bis(azidomethyl)benzene, **1**) (10 mM in DMSO)
Stock solution A2: Bis-alkyne-solution (1,4-Diethynylbenzene, **2**) (10mM in DMSO)
Stock solution A3: Cy7-linker (**38**) (10 mM in DMSO)

Stock solution B: 20 µl TBTA-solution B1 + 10 µl Cu(I)-solution B2
Stock solution B1: TBTA [100 mM in DMSO/*t*BuOH (3:1)]
Stock solution B2: CuI (100 mM in DMSO)*
*must always be freshly prepared and used directly

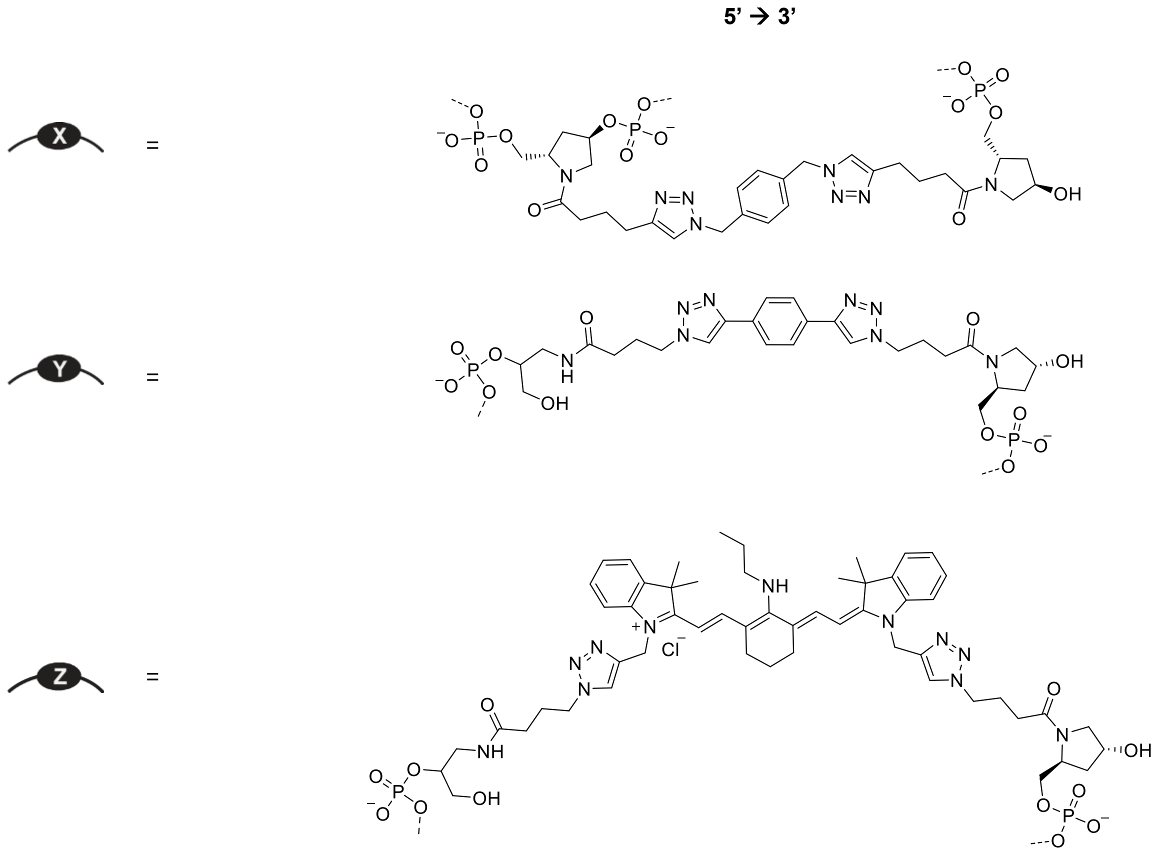


Figure S8: Chemical structure of the connecting linkers X, Y, Z.

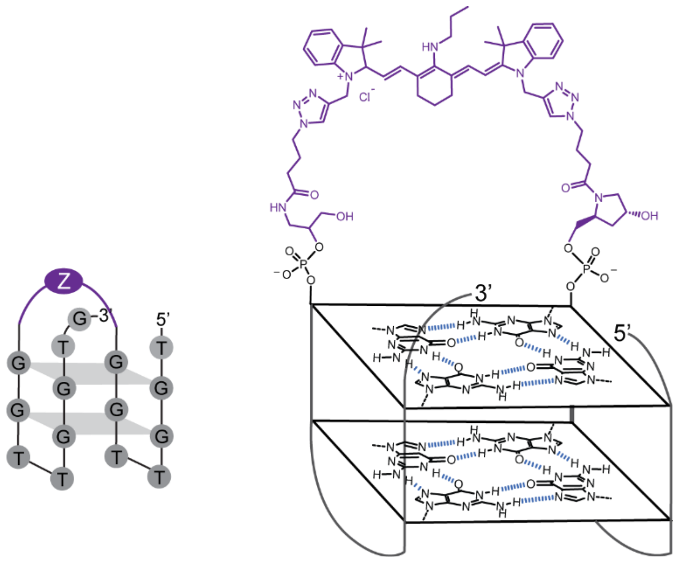


Figure S9: Exemplary schematic representation of the G-quadruplex with conjugated Cy7-linker **8** to spatially illustrate the size relationship.

Table S3: ESI-MS data of the cyclized and stepwise conjugated oligonucleotides used for this study.

| ID | mass calculated [Da] | mass found [Da] |
|---|---|---|
| **B** | 8875.9 | 8876.7 |
| **i-2-bis-alkyne** | 2582.5 | 2582.9 |
| **i-2-Cy7** | 3010.8 | 3010.3 |
| **j-1-azide-Cy7** | 3618.9 | 3617.4 |
| **C** | 5342.1 | 5342.8 |
| **I** | 5770.4 | 5770.1 |
| **J** | 5770.4 | 5770.1 |

compound **B**

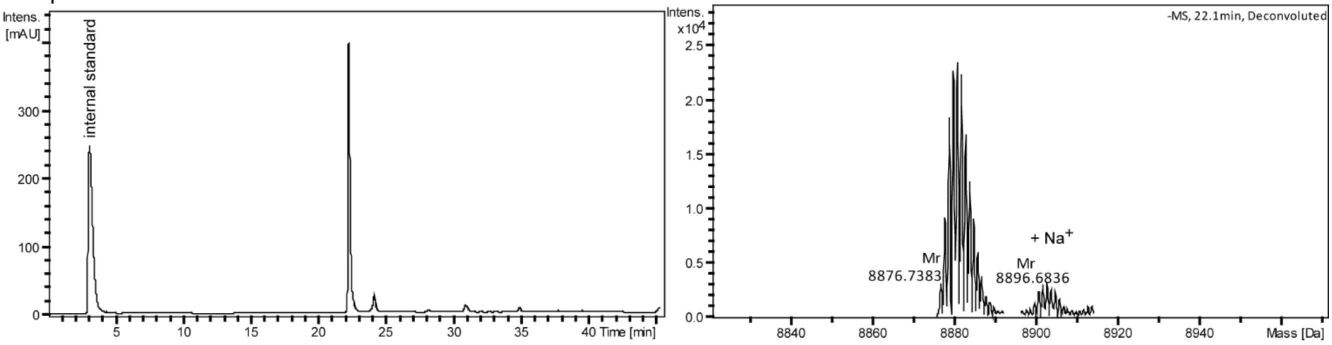


compound **i-2-bis-alkyne**

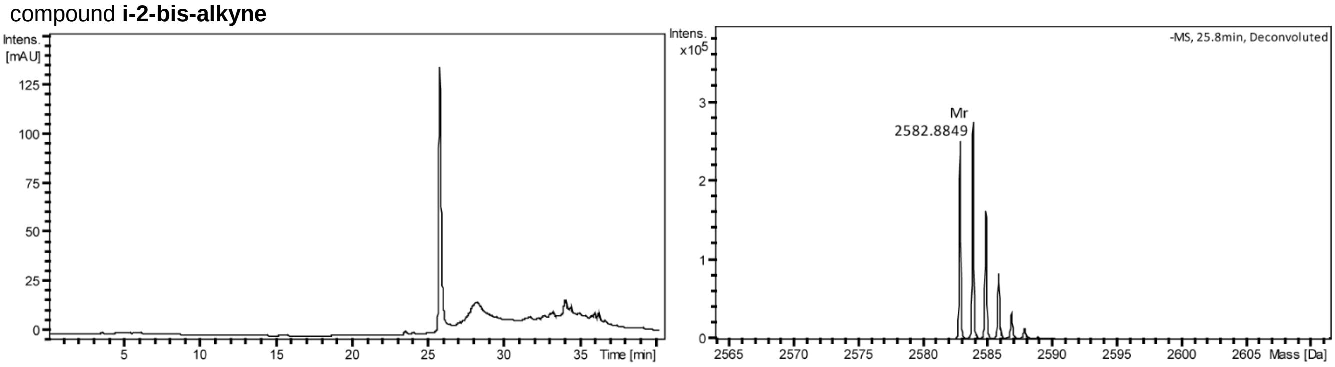


compound **i-2-Cy**

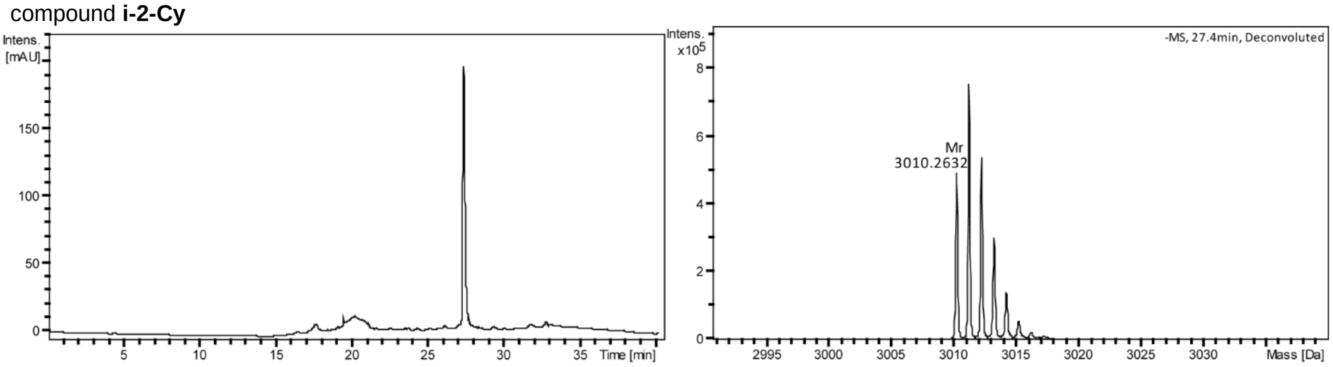

compound **j-1-azide-Cy**

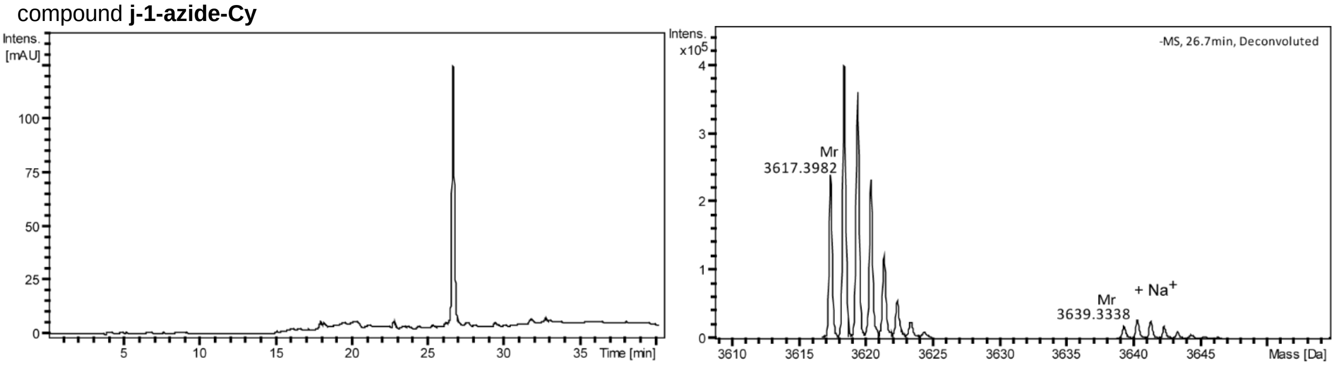


compound **C**

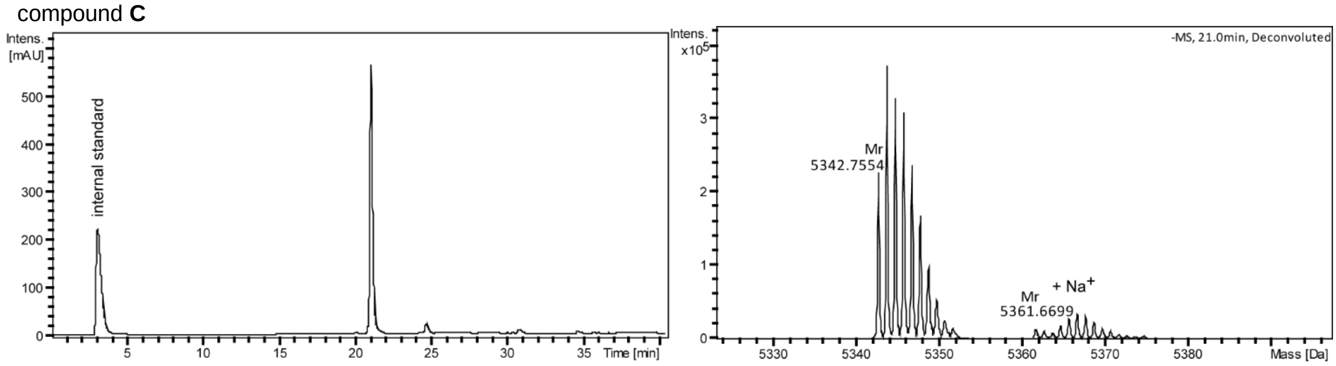


compound **I**

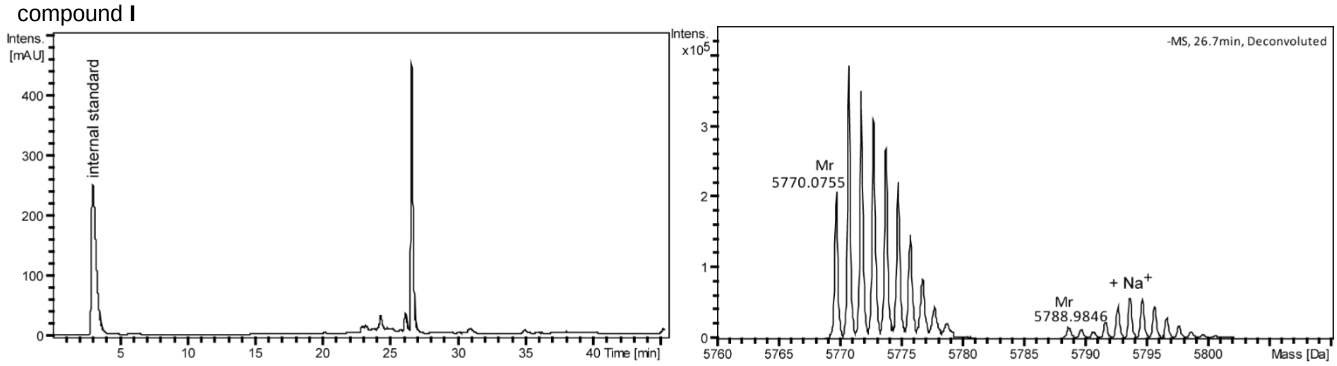


compound **J**

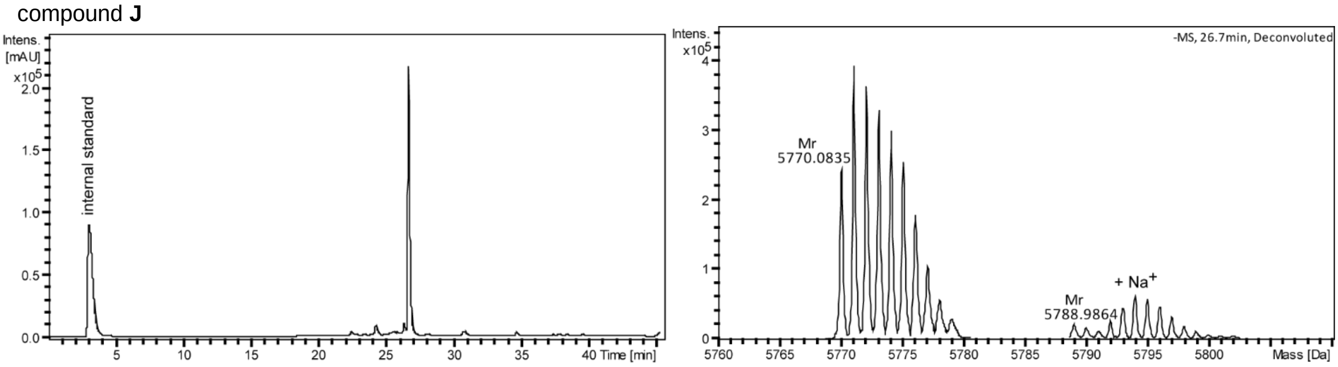


Figure S10: LC-spectrum (left side) and deconvoluted ESI-LC/MS spectrum (right side) of the respective cyclized and conjugated compounds. (LC gradient for **B**: 5% MeOH for 5 min, 5% to 30% MeOH in 2 min, 30% to 80% MeOH in 20 min, 80% to 100% MeOH in 5 min, 100% MeOH for 5 min, 100% to 5% MeOH in 5 min, 5% MeOH for 3 min, flow rate 0.25 ml/min, 40 °C; LC gradient for ***i-2-bis-alkyne***: 5% MeOH for 5 min, 5% to 20% MeOH in 2 min, 20% to 60% MeOH in 16 min, 60% to 100% MeOH in 5 min, 100% MeOH for 5 min, 100% to 5% MeOH in 5 min, 5% MeOH for 2 min, flow rate 0.25 ml/min, 40 °C; LC gradient for all other compounds: 5% MeOH for 5 min, 5% to 40% MeOH in 2 min, 40% to 80% MeOH in 16 min, 80% to 100% MeOH in 5 min, 100% MeOH for 5 min, 100% to 5% MeOH in 5 min, 5% MeOH for 3 min, flow rate 0.25 ml/min, 40 °C).

## 6. Photoproducts

For a description of the photolysis reaction see Schnermann *et al.*[1]

all possible photoproducts for compound **I:**

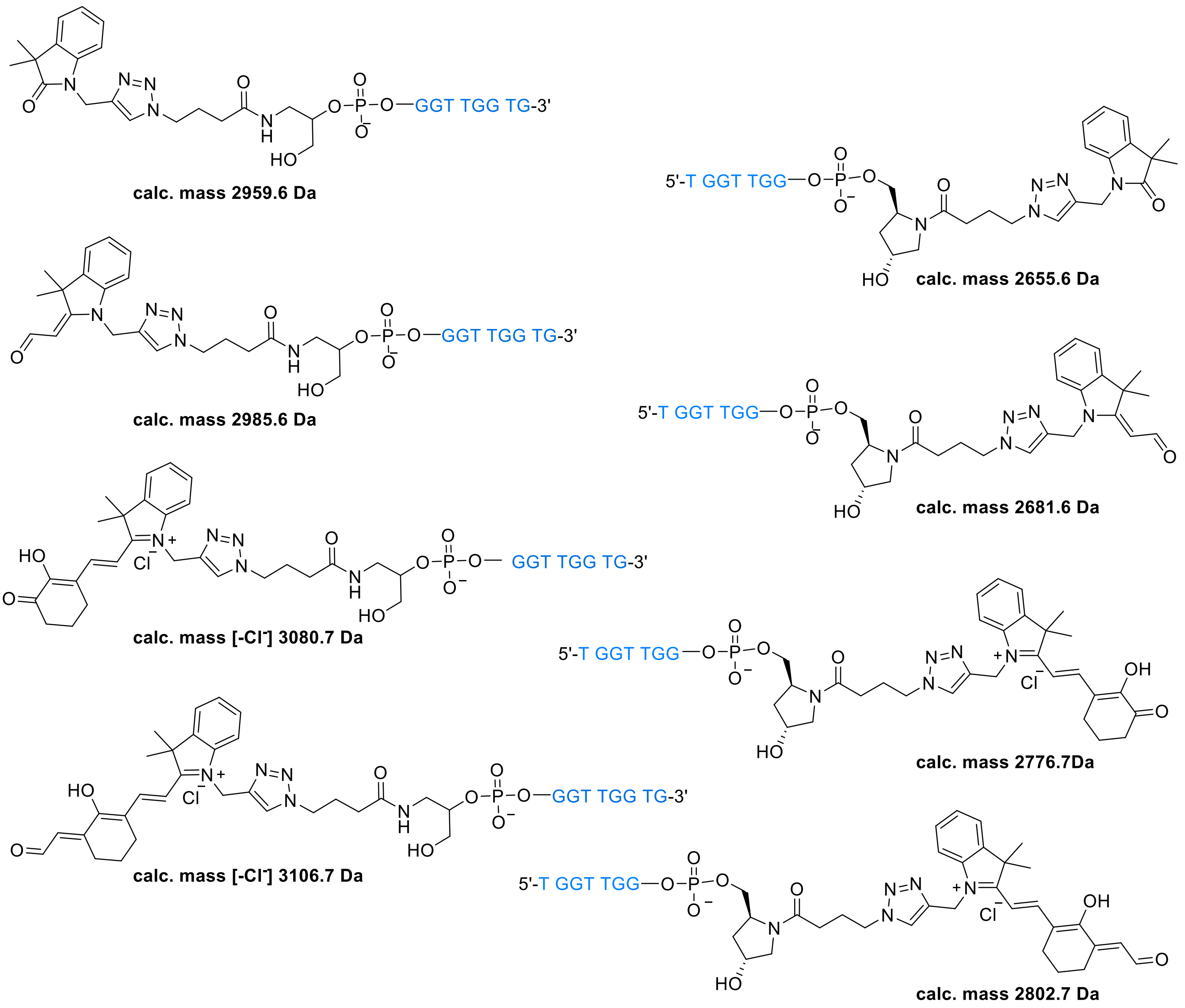


Figure S11: Chemical structures and calculated masses of all purely theoretically possible photoproducts for compound **I**.

Table S4: ESI-MS data of all found photoproducts for compound **I**.

| | Calc. Mass [Da] | Found mass [Da] |
|---|---|---|
| Photoproducts of compound **I:** | | |
| | 2655.6 | 2655.9 |
| | 2681.6 | 2681.9 |
| | 2776.9 | 2775.9 |
| | 2959.6 | 2958.9 |
| | 2985.6 | 2985.0 |
| | 3080.9 | 3079.0 |

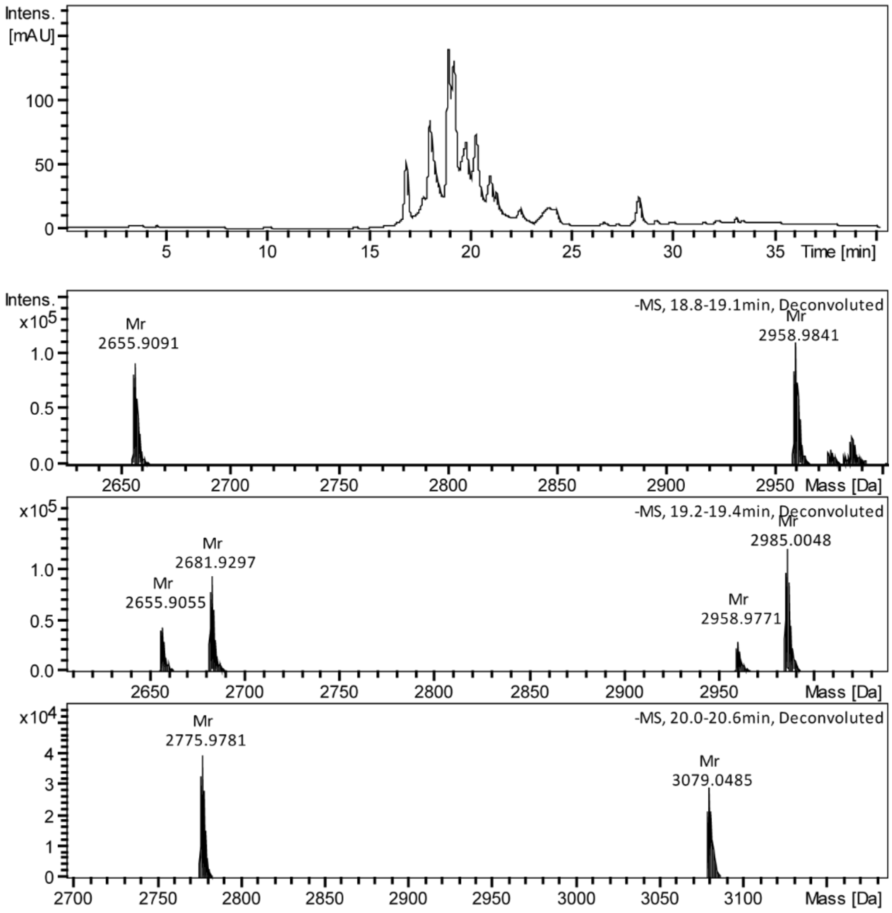


Figure S12: L*C-spectrum (top panel) and deconvoluted ESI-LC/MS spectra of the irradiated compound* ***I****. (LC gradient: 5% MeOH for 5 min, 5% to 40% MeOH in 2 min, 40% to 80% MeOH in 16 min, 80% to 100% MeOH in 5 min, 100% MeOH for 5 min, 100% to 5% MeOH in 5 min, 5% MeOH for 3 min, flow rate 0.25 ml/min, 40 °C).*

all possible photoproducts for compound **J:**

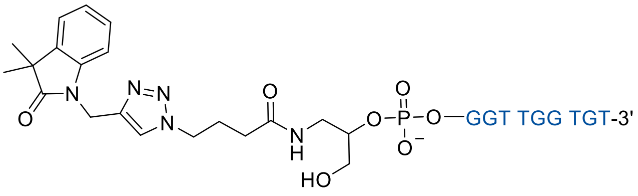

**calc. mass 3263.7 Da**

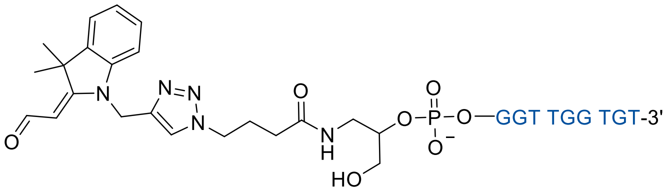

**calc. mass 3289.7 Da**

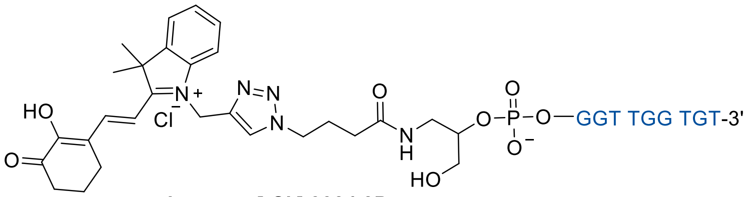

**calc. mass [-Cl$^-$] 3384.8Da**

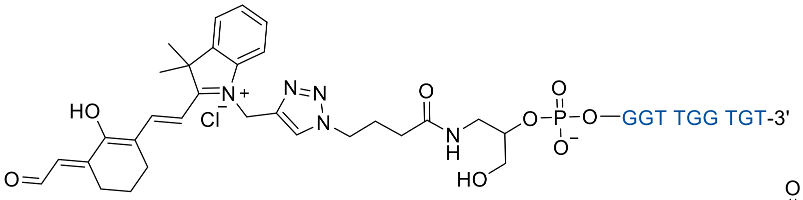

**calc. mass [-Cl$^-$] 3410.8 Da**

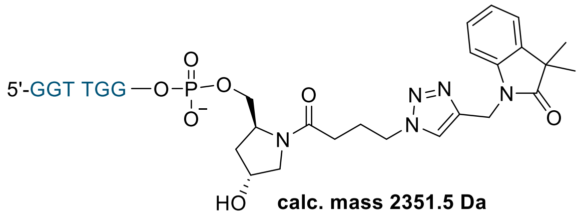

**calc. mass 2351.5 Da**

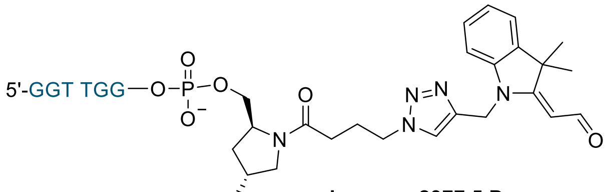

**calc. mass 2377.5 Da**

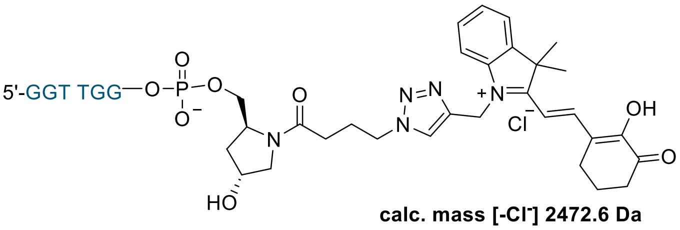

**calc. mass [-Cl$^-$] 2472.6 Da**

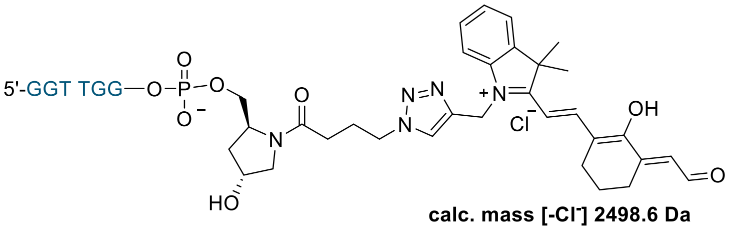

**calc. mass [-Cl$^-$] 2498.6 Da**

Figure S13: Chemical structures and calculated masses of all purely theoretically possible photoproducts for compound **J**.

Table S5: ESI-MS data of all found photoproducts for compound **J**.

| | Calc. Mass [Da] | Found mass [Da] |
|---|---|---|
| Photoproducts of compound **J:** | | |
| | 2351.5 | 2351.9 |
| | 2377.5 | 2377.9 |
| | 3263.7 | 3263.1 |
| | 3289.7 | 3289.1 |

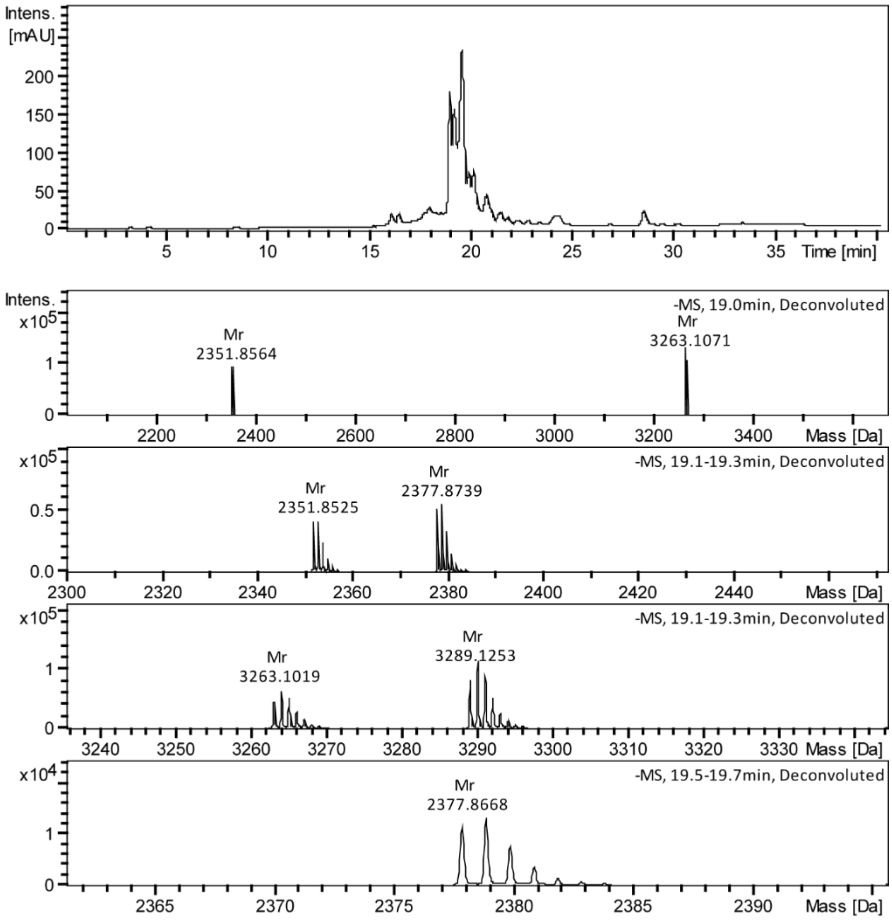


Figure S14: LC-spectrum (top panel) and deconvoluted ESI-LC/MS spectra (right side) of the irradiated compound **J**. (LC gradient: 5% MeOH for 5 min, 5% to 40% MeOH in 2 min, 40% to 80% MeOH in 16 min, 80% to 100% MeOH in 5 min, 100% MeOH for 5 min, 100% to 5% MeOH in 5 min, 5% MeOH for 3 min, flow rate 0.25 ml/min, 40 °C).

## 7. Irradiation

Irradiation of the light-inducible aptamer derivatives was performed with a M660L4 LED (*Thorlabs*, 660 nm, operated at 1000-1200 mA) using a *Thorlabs* LED driver DC2100 or a *Thorlabs* LED driver DC4104 with DC4100-HUB and a ACL 2520-A lens (*Thorlabs*). The exposure time used as well as concentration and buffer are given in the respective chapter.

## 8. CD-spectroscopy

The CD spectra were recorded on a *Jasco J-715* CD spectrometer at 20 °C in a 0.01 cm path length cuvette. For measurements 1 nmol of each oligonucleotide was dissolved in 110 µl of 1xPBS buffer, heated for 5 min to 90 °C, and then allowed to cool to room temperature for at least 1 h until measurement. To measure the irradiated aptamer derivatives, the respective solution was irradiated with a M660L4 LED (*Thorlabs*, 660 nm, operated at 1200 mA, 170 mW) using a *Thorlabs* LED driver DC2100 and a ACL 2520-A lens (*Thorlabs*) for 60 min. Each trace is the average of 5 scans at a scanning speed of 200 nm/min, with a 1 s time constant, 1.0 nm data pitch and 1.0 nm bandwidth. All CD spectra were baseline-corrected for signal contributions of the buffer and smoothed.

## 9. Actinometry

The quantum yield of the Cy7-linker was determined using a fulgide actinometer, according to our previous published protocol.[2] For this purpose, **i-2** was conjugated to both sides of the Cy7-linker **3** using the click protocol described previously, thus obtaining **K**.

Sequence of **K** = 5'-T GGT TGG **m**$^{3}$ – *Cy7-linker* – **m**$^{3}$ GGT TGG T- 5'

**K** was irradiated at 37 °C with a M660L4 LED (*Thorlabs*, 660 nm, operated at 1000 mA, 140 mW) using a *Thorlabs* LED driver DC4104 with DC4100-HUB and a ACL 2520-A lens (*Thorlabs*) in a cuvette (48 µl, d = 3 mm) and durations of 0-1200s. The sample volume consisted of 8 µM **K** and 0.83 µl internal standard (uridine) in PBS buffer. After irradiation, the samples were analyzed by RP-HPLC on a *Agilent 1200* equipped with a *Waters XBridge Peptide BEH C18* column (300 Å, 3.5 µm, 4.6 mm x 250 mm) using gradient 8 (400 mM HFIP/16.3 mM TEA buffer pH 7.9, methanol, gradient 8: 5% MeOH for 5 min, 5% to 40% MeOH in 3 min, 40% to 55% MeOH in 15 min, 55% to 100% MeOH in 3 min, flow rate 0.7 ml/min, 60 °C). Via fulgide actinometry a photon number of 40.47 pmol/s could be measured for the used M660L4 LED. The amount of uncaged **K** was determined by peak integration of the HPLC chromatogram (Figure S15). The initial slope at t=0 was determined by differentiation of the experimental fit of the uncaging kinetics. The quantum yield was then calculated as the ratio between initial slope and the absorbed photon flux. Each experiment was performed in triplicates The calculated quantum yield is $\Phi_p$ = 0.12% and the quantum product is $\Phi_p \cdot \varepsilon_{660}$ = 5.3.

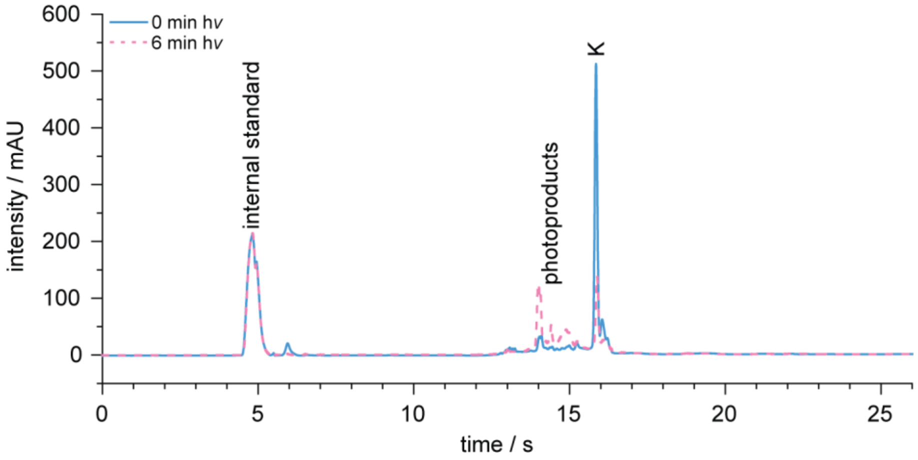


Figure S14: Typical example of a RP-HPLC.

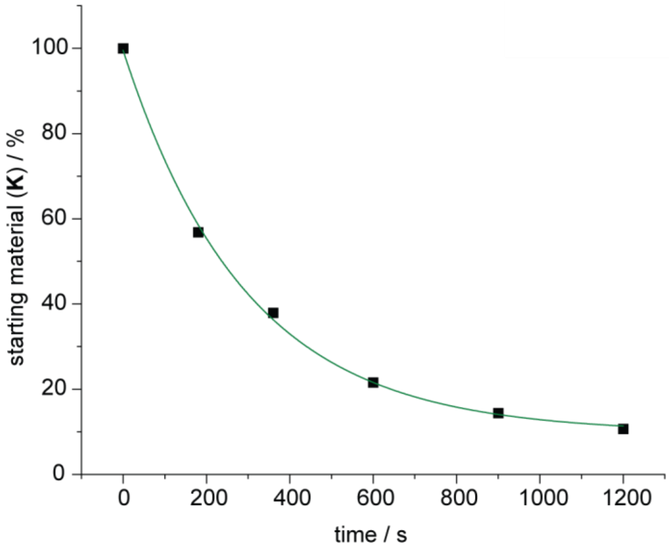


Figure S15: Decrease of starting material **K** as a function of exposure time.

## 10. Clotting assay to measure the thrombin time in human pooled plasma

The anticoagulation activity of the aptamer derivatives was measured in a plasma-based coagulation assay measuring the thrombin time (TT) using an KC 10 A Amelung-Coagulometer. For this, human α-thrombin (CellSystems) was diluted in the assay buffer (1xPBS with 1 µg/µl BSA, pH 7.4) to reach a final concentration of 3 µg/ml. The lyophilized aptamer derivatives were dissolved in Milli-Q water and a dilution series was prepared for each. 50 µl of the thrombin solution was spiked with 50 µl aptamer solution of the respective concentration. After an incubation period of 4 min at 37 °C, 100 µl of pooled human plasma (citrate pooled plasma, in-house preparation, prepared from whole blood taken from healthy blood donors after informed consent, approval of ethics board of the medical faculty of the University Hospital Bonn #070/05) were added and the thrombin time was measured. To measure the anticoagulation activity of the pre-irradiated samples, the light-inducible aptamer derivatives were dissolved 3 µM in HFIP/TEA buffer (400 mM HFIP/16.3 mM TEA buffer pH 7.9) and irradiated with a M660L4 LED (660 nm, *Thorlabs*, operated at 1200 mA, 170mW) using a *Thorlabs* LED driver DC2100 and a ACL 2520-A lens (*Thorlabs*) for 60 min at 37 °C to guarantee the complete deprotection of the photocage and then lyophilized. Each concentration point was measured in triplicates.

## 11. Irradiation of the aptamer-thrombin-complex and fibrinogen coagulation assay

85 µl of a aqueous **TBA**, **I** or **J** solution (0.6 µM) was mixed with 85 µl thrombin solution (0.125 µg/ml in 1x PBS containing 1 µg/µl BSA, pH 7.4) and incubated for 4 min at 37 °C. To measure the irradiated samples, this solution was placed in a cuvette and was irradiated for 30 min at 37 °C with a M660L4 LED (660 nm, *Thorlabs*, operated at 1000 mA, 140 mW) using a *Thorlabs* LED driver DC2100 and a ACL 2520-A lens (*Thorlabs*). Then, the solution was left at 37 °C for another hour. 50 µl of the irradiated or not irradiated aptamer-thrombin-mixture were mixed with 50 µl fibrinogen solution (4 µg/ml, diluted in 1x PBS) respectively in a clear 96 well plate. Absorption at 360 nm was measured on a Tecan Infinite M200 Pro plate reader over 1 h.
To measure the pre-exposed samples **TBA**, **I** or **J** was dissolved 3 µM in HFIP/TEA buffer (400 mM HFIP/16.3 mM TEA, pH 7.9) and irradiated for 30 min at 37 °C with a M660L4 LED (660 nm, *Thorlabs*, operated at 1000 mA, 140 mW) using a *Thorlabs* LED driver DC2100 and a ACL 2520-A lens (*Thorlabs*). After another hour at 37 °C, the samples were lyophilized. To measure the activity, the respective sample was dissolved in Milli-Q water and treated in the same way as described above. Each sample was measured in triplicates.

## 12. Irradiation in human whole blood and coagulation assay

80 µl human citrate-blood (from healthy blood donors after informed consent, approval of ethics board of the medical faculty of the University Hospital Bonn #070/05) was spiked with 20 µl of **TBA** or **I** solution (2 µM in Milli-Q water). For irradiation, the solution was placed between two petri dishes (d=0.5 mm), with the upper one filled with water to form a thin layer of water. The irradiation was performed with a M660L4 LED (660 nm, *Thorlabs*, operated at 1200 mA, 350 mW) using a *Thorlabs* LED driver DC2100 and a ACL 2520-A lens (*Thorlabs*) over different time periods.
To measure the activity, 60 µl of the respective irradiated or not irradiated blood-aptamer-mixture (or as a control blood-water-mixture) were placed in a PCR tube and heated to 37 °C over 2 min, then 10 µl thrombin solution (1 µg/ml in 1x PBS containing 1µg/µl BSA, pH 7.4) were added and incubated over 2 min at 37 °C. After 2 min at room temperature, the PCR tubes were turned upside down, tapped and then the photo was taken. Temperature changes due to irradiation were < 4°C.

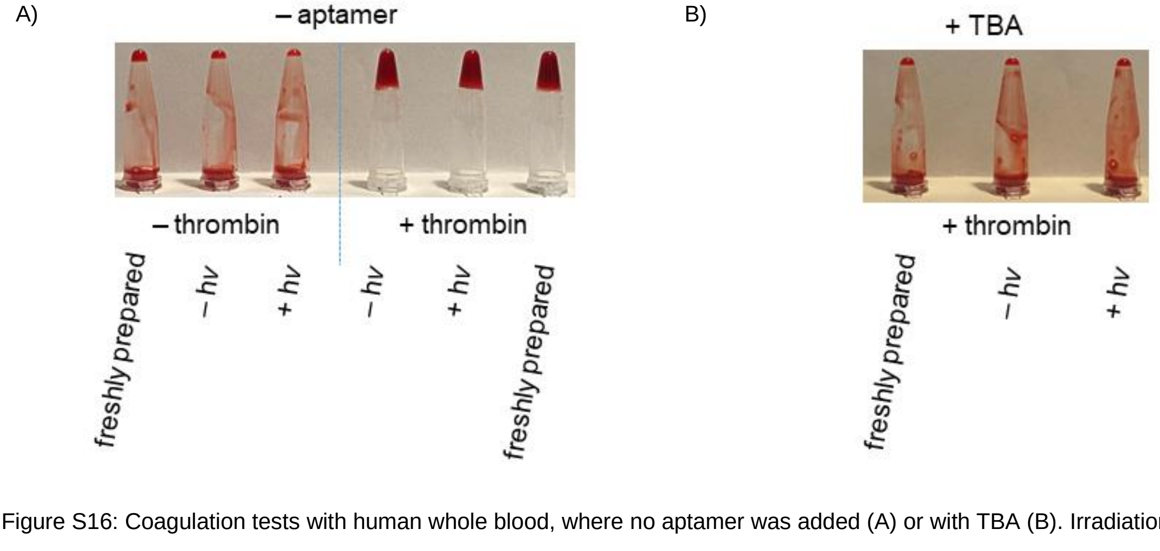


Figure S16: Coagulation tests with human whole blood, where no aptamer was added (A) or with TBA (B). Irradiation time = 20 min.

## 13. Plasma Stability

10 µl oligonucleotide solution (25 µM in Milli-Q water) were mixed with 40 µl human pool plasma. The resulting assay mixture was incubated at 37 °C for a total of 24 hours. Samples were taken at different time points as indicated in the figures. The degradation process was stopped by mixing the sample (4 µl of the assay mixture) with 6 µl gel-lading buffer (80% 5 M urea in formamide, 8% 500 mM EDTA in water, 12% formamide), heated to 95 °C for 3 min, and then stored at – 20 °C. The subsequent analysis was carried out by gel-electrophoresis on 20 % denaturing polyacrylamide gels using 7 M urea in 1xTBE (Tis-borate-EDTA) as running buffer. The gels were run at room temperature for 40 min at 200 V, stained with a SYBRGold solution and visualized with a gel imager (*BioRad*, GelDoc XR+).

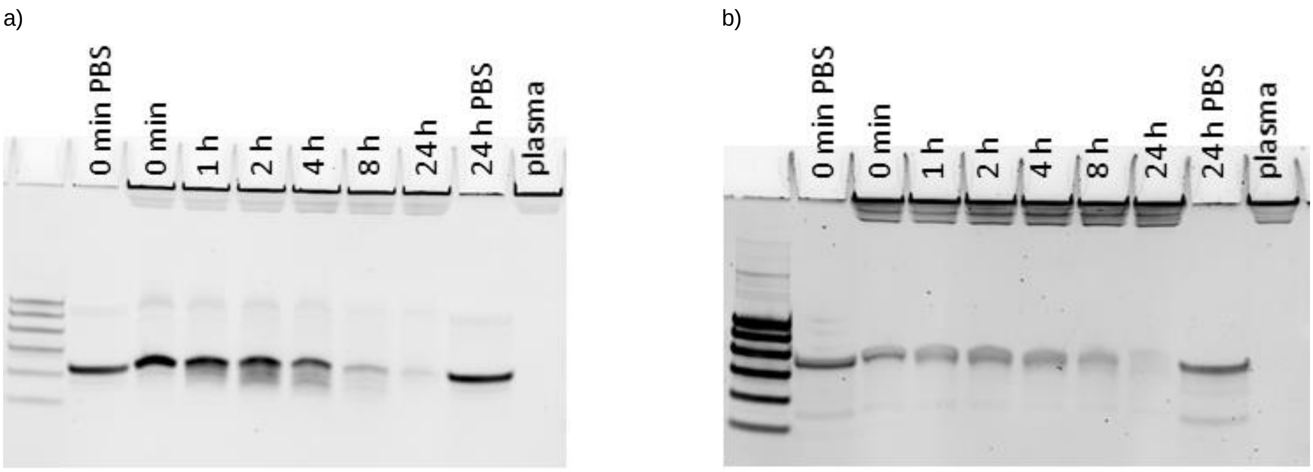


Figure S17: 20 % denaturing polyacrylamide gels for analysis of stability of **I** (a) and **TBA** (b)in human pool plasma. Incubation in 1x PBS buffer served as control.

## 14. Literature

[1] A. P. Gorka, R. R. Nani, J. Zhu, S. Machem, M. J. Schnermann, *J. Am. Chem. Soc*. **2014**, *136*, 14153-14159.

[2] M. Reinfelds, V. Hermanns, T. Halbritter, J. Wachtveitl, M. Braun, A. Heckel, *ChemPhotoChem*. **2019**, *3*, 441-449

## 15. List of abbreviations

| | |
|---|---|
| ac | acetyl |
| BTT | 5-(Benzylthio)-1*H*-tetrazol |
| CE | cyanoethyl |
| CuAAC | copper catalyzed alkyne-azide-cycloaddition |
| DMT | 4,4‘-dimethoxytrityl |
| EDTA | ethylenediaminetetraacetic acid |
| Fmoc | fluorenylmethyloxycarbonyl |
| HFIP | 1,1,1,3,3,3-hexafluoro-2-propanol |
| iPr | isopropyl |
| MeCN | acetonitrile |
| NHS | *N*-hydroxysuccinimide |
| pac | phenoxyacetyl |
| PBS | phosphate buffered saline |
| tac / tBPAC | 4-tert-butylphenoxyacetyl |
| TEA | triethylamin |
